# A remote sensing-based survey of archaeological/heritage sites near Kandahar, Afghanistan through publicly available satellite imagery

**DOI:** 10.1371/journal.pone.0259228

**Published:** 2021-11-02

**Authors:** Mehmet Karaucak, Daniel Steiniger, Nikolaus Boroffka

**Affiliations:** Deutsches Archäologisches Institut, Eurasien Abteilung, Berlin, Germany; University at Buffalo - The State University of New York, UNITED STATES

## Abstract

Due to its conflict-ridden recent history, it has been difficult to launch major archaeological projects and advanced field research in Afghanistan during the last forty years. Lately, the proliferation of remote sensing methods, especially the increasing availability of satellite imagery, has allowed generating a much-needed impetus for documentation and monitoring of the heritage in Afghanistan. In this study, we present novel site data obtained through an examination of publicly available satellite imagery in the southwestern region of Kandahar. The sites presented here consist of a multitude of cultural heritage such as settlement mounds, architectural remains, religious monuments, fortresses, and traditional water management systems. We also discuss the advantages, as well as the drawbacks of remote sensing surveys for archaeological research in Afghanistan, and share our data to be employed in further research and cultural heritage management in the region.

## Introduction

Afghanistan has been in a constant state of war during the last four decades. After the beginning of the Civil War in 1978, the conflict in Afghanistan has evolved in many directions, becoming ever increasingly complex. While the actors and alliances involved in the war continually changed and transformed throughout the course of these forty years, each shift in the power dynamics has added to the complexity and endurance of war. The plight of war that befell civilian populations, though, remains unchanged. Besides human suffering, an unprecedented attack on Afghanistan’s cultural heritage has unfolded in broad daylight, sometimes even on live TV. While high-profile attacks, such as the notorious case of the Bamiyan Buddha [1], have captured the attention of the international community, the true size and scale of the war-related damage, as well as the effects of sustained looting of cultural heritage are still under assessment [2–6].

Climate change is the other major factor that contributes to the overall deterioration of the human condition in Afghanistan, and especially in the sensitive dryland regions around the Registan Desert and southern Afghanistan. Intensely arid conditions, which began in 1998, have led to the displacement of around 100.000 nomads who used to live and stock breed in the Registan Desert [7]. The ongoing state of conflict, coupled with the worsening climatic conditions, has considerable implications on archaeological research and the preservation of cultural heritage in Afghanistan. Field research, due to security reasons, is not possible in large parts of the country where government control is either minimal or non-existing. For this reason, archaeological research in Afghanistan may benefit from the use of alternative methods and data sources.

Satellite technology provides a means to remotely capture multi-spectral data from space, including spectral reflectance and emissivity that is invisible [8, 9]. There has been a significant increase in the accessibility of satellite imagery following the declassification of the CORONA program by the US government in 1992. The capability to remotely observe mounded sites as well as the ancient levees that supported them with water had a profound impact on Southwest Asian archaeology, particularly on the question of urbanism and the emergence of first cities [10–23]. The use of satellite imagery and aerial photography in archaeology was not limited to the Southwest Asia. Similar methods were applied elsewhere on entirely different research themes: Such as the case of Tripolye mega-sites of the 4^th^ Millennium BC in the northwestern Pontic Region [24, 25], Bronze Age geoglyphs in Kazakhstan [26, 27], or the Polynesian geoglyphs in New Zealand and the Pacific which are dated before the European colonization of the islands [28]. Currently, there is a growing body of literature on the use of satellite data for site detection [29–32], or on obtaining data on past land-use [33], cultural heritage management [34–39], and more recently on ancient mining and metallurgy [40]. The number of studies dealing with the archaeology of Afghanistan and cultural heritage management through satellite imagery has also been increasing in recent years [6, 41–43].

## Study area

**Fig 1:**
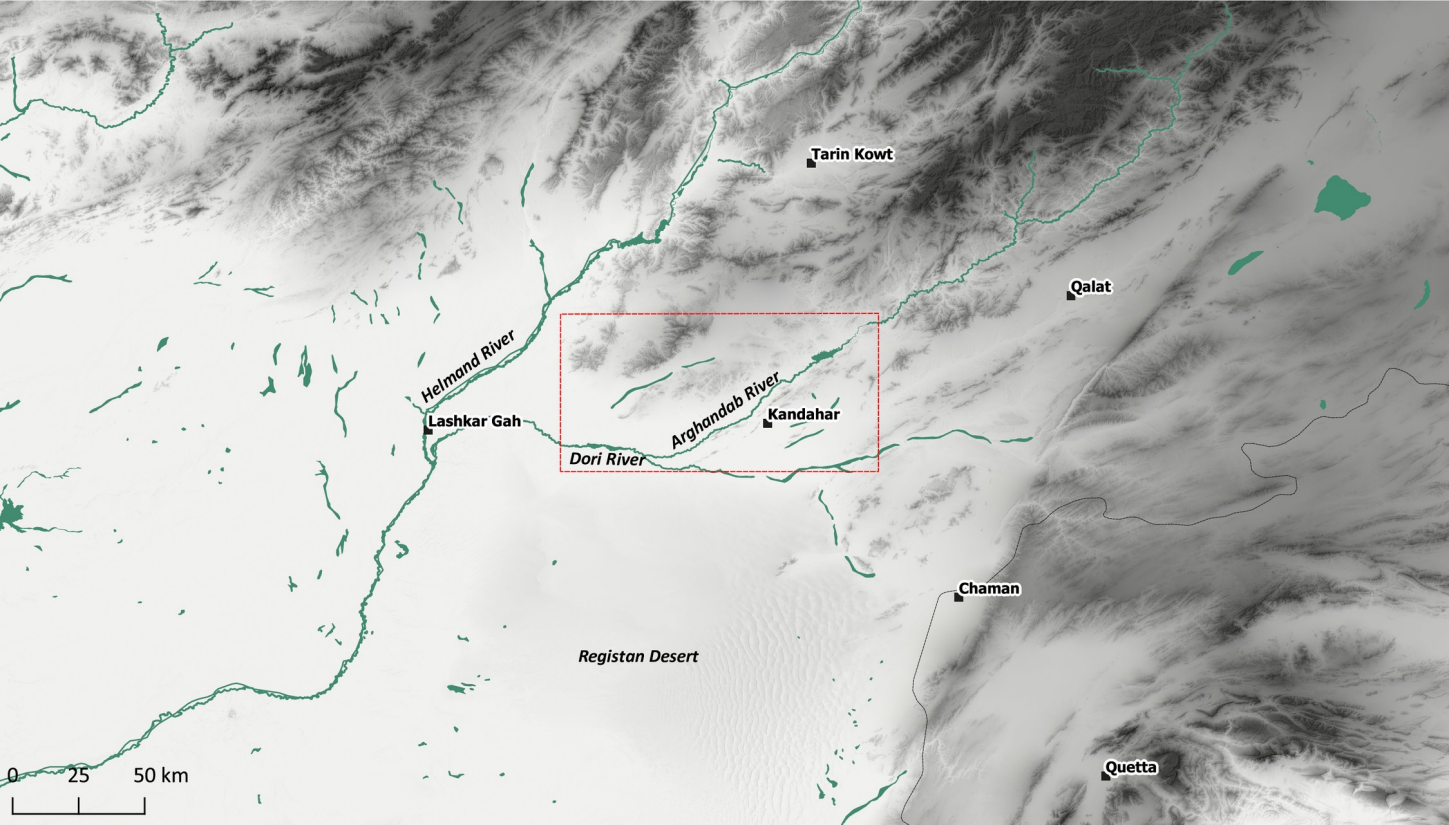
Map of the Kandahar region and Registan Desert. DEM based on Shuttle Radar Topography Mission (SRTM) 1 Arc-Second Global courtesy of the U.S. Geological Survey (doi:10.5066/F7PR7TFT) (https://earthexplorer.usgs.gov). The map was created by the authors in QGIS.

The area around Kandahar, which has been selected in this study to be surveyed for archaeological points of interest, consists of a diverse terrain configuration and encompasses multiple micro-climate zones (Fig 1). Kandahar is situated at the southern tip of the Hindu Kush Mountains, on a broad alluvial fan that is mainly drained by the Arghandab River and its tributary Tarnak. The immediate surroundings of Kandahar are classified as semi-arid (according to the Köppen Climate Classification), suggesting irregular and low rates of rainfall and high variation between summer and winter temperatures. Winters in Kandahar are usually harsh and cold, with freezing temperatures that occur mostly during the night time. Springs are unpredictable, although this is the season when water is most abundant due to the melting snow in the mountains. Summers and autumns, in contrast, are the driest seasons in the region [44]. The extreme scarcity of water is a factor that greatly constrains the growth and diversity of natural vegetation. The most common plants to be found in Kandahar province are desert grasses, shrubs, and thistles that are specifically adapted to thrive in such arid environments [44, 45].

The study area presented here consists not only of the immediate surroundings of Kandahar but also extends to the mountain valleys to the north of the city, as well as a narrow strip of the Registan Desert to the south. Therefore, a gradient of variation in climate and vegetation is apparent between the desert landscape of the south, the lush alluvial zone, and the barren hills to the north. Regrettably, the past climatic conditions in the region are completely understudied. Nevertheless, detailed paleoclimate research in the Lake Hamoun area in the borderland between Afghanistan and Iran (near the prehistoric site Shahr-i Sukhteh) has revealed several conclusions [46] that can be used as an approximation for the study area, although the micro-climate conditions of each region were individual. On a supra-regional scale, the dominant wind circulation systems which influenced the regional climate in the Helmand basin, with Shahr-i Sukhteh in the westerly direction and Kandahar in the northeast, were affected during the early Holocene by the Indian summer monsoon while the present-day monsoon does not reach further northwest than the Indus valley, due to a southeasterly shift of the inter-tropical convergence zone through time. Therefore, a convenient climatic phase seems to have facilitated the spread of Chalcolithic and Bronze Age settlements in the Helmand basin (and Balochistan before the so-called ‘4.2 event’, a dry phase around 4200 BP) and a climatic optimum can be reconstructed also most likely during Achaemenid and Sassanian times. Different climatic regimes were dominant for instance in northern Afghanistan and Central Asia (Siberian anti-cyclone system), in Central Iran (mid-latitude westerlies), and in the Indus valley (Indian summer monsoon), for which reason paleoclimate reconstructions for each broader region reveal peculiar features in space and time, including correlating effects on water supply and human settlement distribution [46–48]. However, at present, we have almost no data to reconstruct the particular environmental setting of Kandahar and its surroundings during prehistory. Nonetheless, the presence of Sassanian and Early Islamic settlements in the Registan Desert [44, 49, 50] suggests that even the most arid parts of the region could have been more suitable for human life in the past.

The main watercourse in the region, the Arghandab River, flows southwest towards the Helmand River. It is the most important source of water for the city of Kandahar and its surrounding villages. Irrigation in the region, today, is mostly supported by the Dahla Dam–the only major dam in the province–while underground water sources are also being increasingly exploited for agricultural production. The traditional surface canals and *qanats* (or *karez* in Pashto), which combine a series of shafts and underground tunnels, still play an important role in the irrigation of fields, vineyards, and orchards. In the desert regions to the south, wells and *nawars* (traditional water reservoirs that collect runoff water) provide the water supply [7]. The utilization of bore-wells coupled with mechanical diesel pumps has increased rapidly in the last ten years due to the severe drought conditions that the region has been experiencing, putting further strain on the groundwater sources.

Although it is very limited, past research provides evidence of the region being continuously populated at least since the 4^th^ Millennium BC. The excavations at the earlier levels of Mundigak, Said Qala Tepe and Deh Morasi Ghundai have revealed village sized settlements that were occupied throughout the 4^th^ and 3^rd^ millennia BC, and that some of them gradually developed into larger and more elaborate towns over the course of the 3^rd^ Millennium BC [51, 52]. The presence of monumental public/elite architecture in Mundigak [53], as well as the circulation of metals and semi-precious stones such as *lapis lazuli* [54–60], are suggestive of complex social organization and participation in the long-distance exchange networks during the Bronze Age.

As an urban centre, Kandahar played an important regional role during the Achaemenid Period, serving as the capital city of Arachosia before it fell to the armies of Alexander around 330 B.C., and its eventual renaming as Alexandropolis. The Greek rule in Arachosia was, however, short-lived. The city, like the rest of the region, was conquered by the Mauryan Empire, probably during the rule of Aśoka by the middle of the 3^rd^ Century B.C. Around 190 B.C., Arachosia was once again brought under Greek rule by the Graeco-Bactrian king Demetrius I. This final phase of Greek rule in the city lasted until its conquest by the nomadic Śaka, sometime during the first half of the 1^st^ Century B.C. [61]. Kandahar’s importance as a trade hub, owing to its location between India and Herat, has long been stressed [62]. Greek texts mention a land route between Mesopotamia and Central Asia that ended in Kandahar [63, 64], from where further routes towards the northeast to Gandhāra and Taxila or the southeast to the lower course of the Indus River could have been taken [65, 66].

We have chosen to focus on the Kandahar region in this study primarily due to its geographic characteristics with the expectation of locating settlement mounds that are considered typical for such well-drained lowland basins across much of the Southwest and Central Asia. The presence of an early urban centre in Kandahar underscores this agricultural potential that the region could have offered the sedentary societies which choose to settle here in the past. The region is also quite rich in mineral resources, with many known outcrops of copper, tin, and gold across the Hindu Kush foothills to the north, while the presence of an early metal industry has already been attested during the excavations at settlement sites like Mundigak, Said Qala Tepe and Deh Morasi Ghundai. When taken into consideration with the interconnected nature of this region with distant prehistoric centres in Iran, Mesopotamia, and the Indus Valley, these conditions hint at the high potential of Kandahar for further archaeological research.

## Materials and methods

### Data sources

In this study, we make use only of high-resolution satellite imagery from publicly available online sources and open-source software to survey the Kandahar region for potential archaeological sites. There are several online sources which provide satellite imagery with local or global coverage. The data from these sources are usually provided as individual tiles that can be downloaded, or in the form of a Web-map service (WMS) that can be accessed, or ’streamed’ directly from the service provider’s dedicated server. For this study, we have preferred to use the WMS of Google and ESRI as basemaps, both of which are publicly accessible. These basemaps provide adequate resolution and detail for this study. They are appropriate to evaluate the general characteristics of the region as well as to locate the sites that were already known through previous research and to survey the area to locate new sites. Both sources can be accessed and viewed without a paywall. The images were viewed in the QGIS platform (v.3.4), which is an open-source and freely distributed software package. QGIS allows users to establish a connection to active WMS servers through its layer settings so that the data on the WMS server can be downloaded and viewed in real-time as an individual layer within a GIS project. The terrain models and elevation data used in the study are based on Shuttle Radar Topography Mission (SRTM) data and irrigated land data is provided by the United Nations Environment Program.

The Archaeological Gazetteer of Afghanistan by Warwick Ball, published first in 1982, and its revised 2019 edition provide a comprehensive catalogue of previously recorded archaeological sites in Afghanistan, as well their approximate geographic coordinates. In its revised edition, the gazetteer contains information about more than 1500 sites across Afghanistan which are dated between the Palaeolithic and the Timurid period. It is also the only such comprehensive resource for the region under consideration here, and accordingly, it serves as the archaeological basis and starting point for our study.

### Methodology

Aerial photography and satellite imagery are the two main applications of remote sensing in archaeology. Geophysical survey methods such as magnetometry, electrical resistance, and ground-penetrating radar will not be taken into consideration here, although they are also included within the constellation of archaeological remote sensing methods. Aerial photography can be regarded as the more established of these two methods, since it has been available for a longer time, and the essentials of site detection were established through its utilization. It has found many applications in archaeological research since the end of the 19^th^ Century, with increasing frequency after World War I due to the rapid technological advances in the fields of photography and flight engineering [67]. Following the proliferation of drone technology during the last two decades, aerial photography has become an integral part of archaeological fieldwork since it provides the most accurate and cost-effective way of documentation for both surveys and excavations [see 68 for an introduction and overview of drone-based survey methodologies in landscape archaeology].

Different approaches in the application of satellite imagery for archaeological and cultural heritage studies have been suggested in recent years. There have been attempts to automate the survey process by developing and applying rule-based site detection protocols [40, 69–74], or by utilizing artificial intelligence and machine learning [75]. Other researchers have outlined ‘crowd-sourced’ approaches that depend on the contribution of a large number of participants for the detection of archaeological features [76, 77]. Nevertheless, the conventional methods (manual, non-automated, non-crowd-sourced visual inspection) of archaeological site detection are still widely applied in archaeological site detection. Although much slower in comparison with the automated or crowd-sourced analysis of satellite imagery, the manual detection provides more nuanced and better-contexted results by entailing an interpretative dialogue with data at each step of the study [79]. Another reason for our preference to manually locate sites is the general lack of existing information that could have been used as a template to formulate a rule-based procedure for automated site detection.

The survey method applied in this study relies on the visual inspection and interpretation of photographic data collected by satellites. To systematize this process, the study area (118 x 68 km) was delineated as a vector and divided into a grid consisting of 0.01° x 0.01° (~1.1 x 0.95 km) tiles. Each tile was inspected separately on both Google and ESRI basemaps, and the features were saved as points. Each point was labelled with a four-digit code, starting with the letter “K” which stands for “Kandahar”. We have separated the sites that are in the gazetteer of Warwick Ball [56, 57] by starting their labels from “K000”, while the new sites were labelled starting from “K100”. The information related to these features; such as their type, terrain characteristics, ground plan or form, length, width, area, and any visible signs of physical damage were compiled using the attribute table module built into QGIS.

## Results

### Mounds

Settlement mounds are stratified multi-period settlement sites that form as a result of long-term human occupation in the same location. The accumulation of settlement refuse and construction materials through repeated actions of building, destruction, and deposition, together with the influence of natural forces such as wind/rain erosion/deposition leads to the formation of distinctive hill-like topographic features in the landscape. The settlement mounds which have already been investigated by excavations and ground-based surveys provide a good reference point for the visual qualities of mounds in the region. Among the known mounded sites in Kandahar, Spirwan [K002; 56, 57, site codes: 1982; 2019:1109] is the most conspicuous one due to its large surface area and its steep flanks. Its irregular shape, as well as the remains of a citadel on its western eminence, provide distinctive elements that help distinguish Spirwan from the surrounding landscape of cultivated fields and small farmsteads. The other investigated mounds in the region, such as Deh Morasi Ghundai [K003; 56, 57, site code: 287], Said Qala Tepe [K001; site code: 968], and Mundigak [K019; site code: 743], are much smaller in comparison with Spirwan, and therefore their topographic features are less pronounced. These sites also lack some of the distinctive visual elements present in Spirwan’s case, and therefore they blend in much better with their surroundings. Nevertheless, when viewed in the 1/2000 scale, a distinctive change in soil colour is discernible in all three cases. Although their topographic qualities are far subtler, it is still possible to distinguish these smaller mounds by their surface colour and texture.

The region under study consists mostly of rocky hills and flat plains among the valleys. While mounded sites are fairly easy to spot on flatlands with relatively sparse topographic features, the valleys and plains around Kandahar contain many high ridges and rock outcrops. In some cases, the surface texture of the feature can be helpful to distinguish mounds from rock outcrops, as the surface of the mound seems often more homogeneous and duller due to the fine sediments it consists of. The presence of visible architectural elements such as traces of wall foundations on the surface is also a good indicator. The surface of the mounds that are situated within sown fields are most often left uncultivated in the Kandahar region and therefore such cases are much easier to distinguish in comparison with the mounded sites in drier and sparsely vegetated areas.

**Fig 2:**
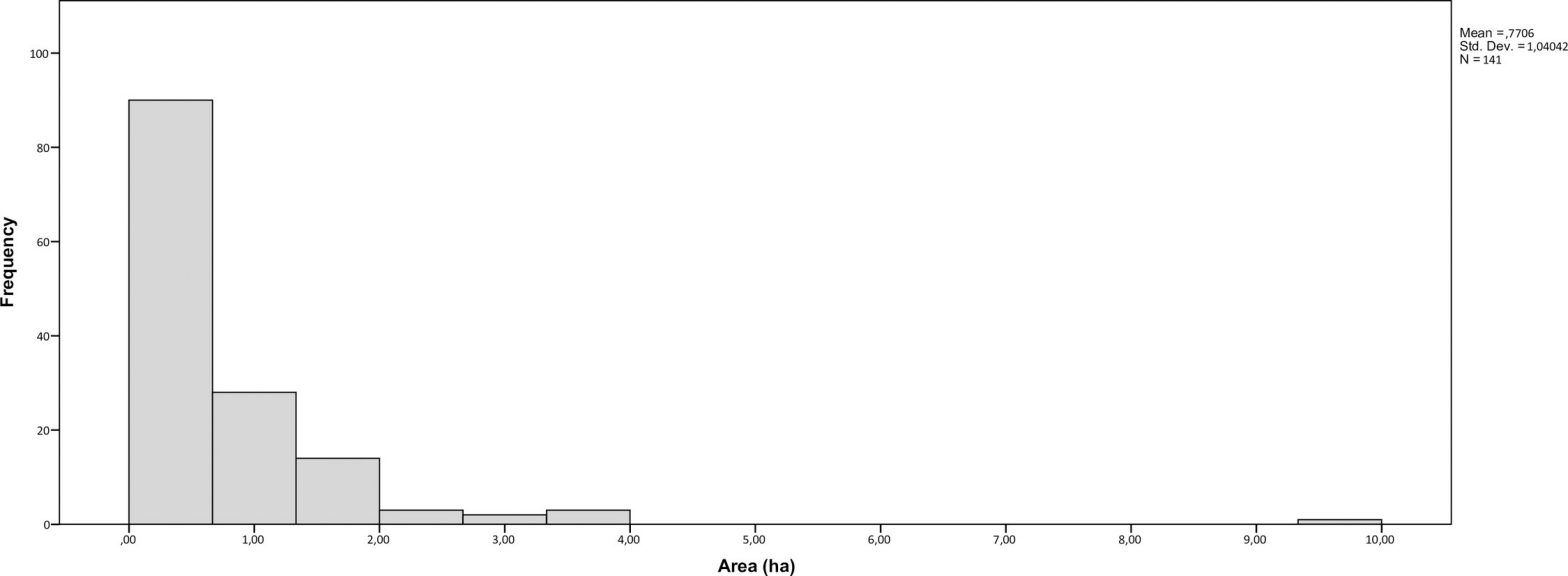
Histogram showing the surface area range of mounded sites.

Ten settlement mounds dated to various periods between the Chalcolithic and the Medieval were known previously in the Kandahar area [56, 57, site codes: 85; 180; 287; 743; 754; 798; 968; 976; 1109; 1249]. During this study, we were able to observe 138 new possible mounded sites in the region. The mounds in Kandahar are diverse in terms of their size, as their surface areas range from 0.05 ha to 9.81 ha (Fig 1). Accordingly, we think that while the larger mounded sites (>0.35 ha) may represent settlement mounds, the smaller ones can be interpreted in various ways such as *kurgans*, stupas, or eroded remains of single structures. In this sense, 90 cases can be interpreted as potential settlement mounds since their surface area exceeds 0.35 ha (Fig 2).

**Fig 3:**
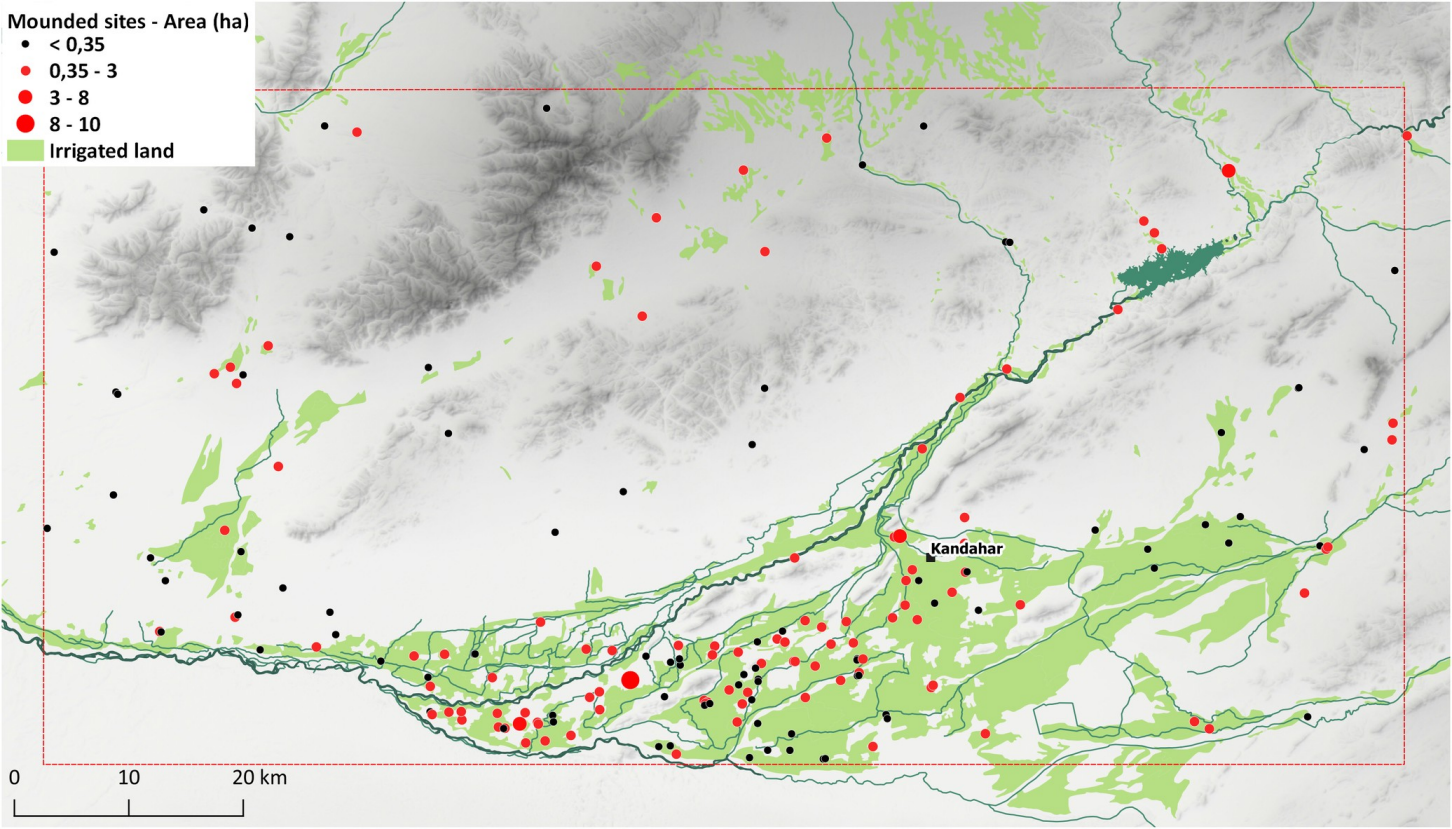
Map showing the distribution of mounded sites. DEM based on Shuttle Radar Topography Mission (SRTM) 1 Arc-Second Global courtesy of the U.S. Geological Survey (doi:10.5066/F7PR7TFT) (https://earthexplorer.usgs.gov). The map was created by the authors in QGIS.

**Fig 4:**
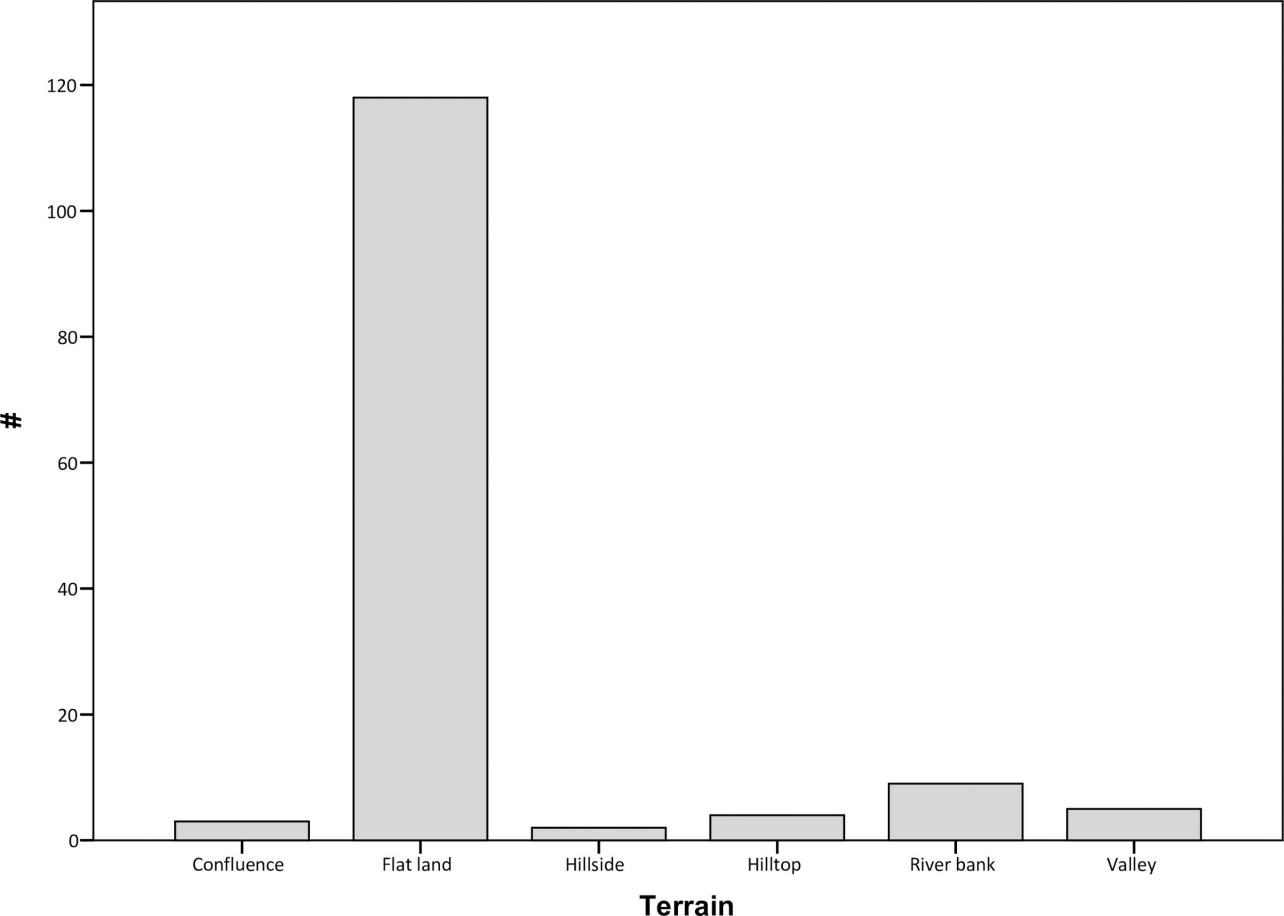
Graph showing the distribution of mounded sites according to terrain type.

The distribution of mounded sites in Kandahar region is to a large extent bound by the arable lands and the availability of freshwater sources. Except for 27 sites, all mounded sites are located within the alluvial zone between the Arghandab river and its tributaries Tarnak, Dori and Arghastan. The flatlands, slightly far from river banks, seem to be the preferred locations for mounds, with the vast majority of sites being distributed in such terrain. Riverbanks, valleys and hills are, in contrast, less densely occupied (Figs 3 and 4). There is also a weaker correlation between the extent of currently irrigated areas and mound distribution, with 80 sites located within these zones. This might be indicative of different water management strategies in the past.

**Fig 5:**
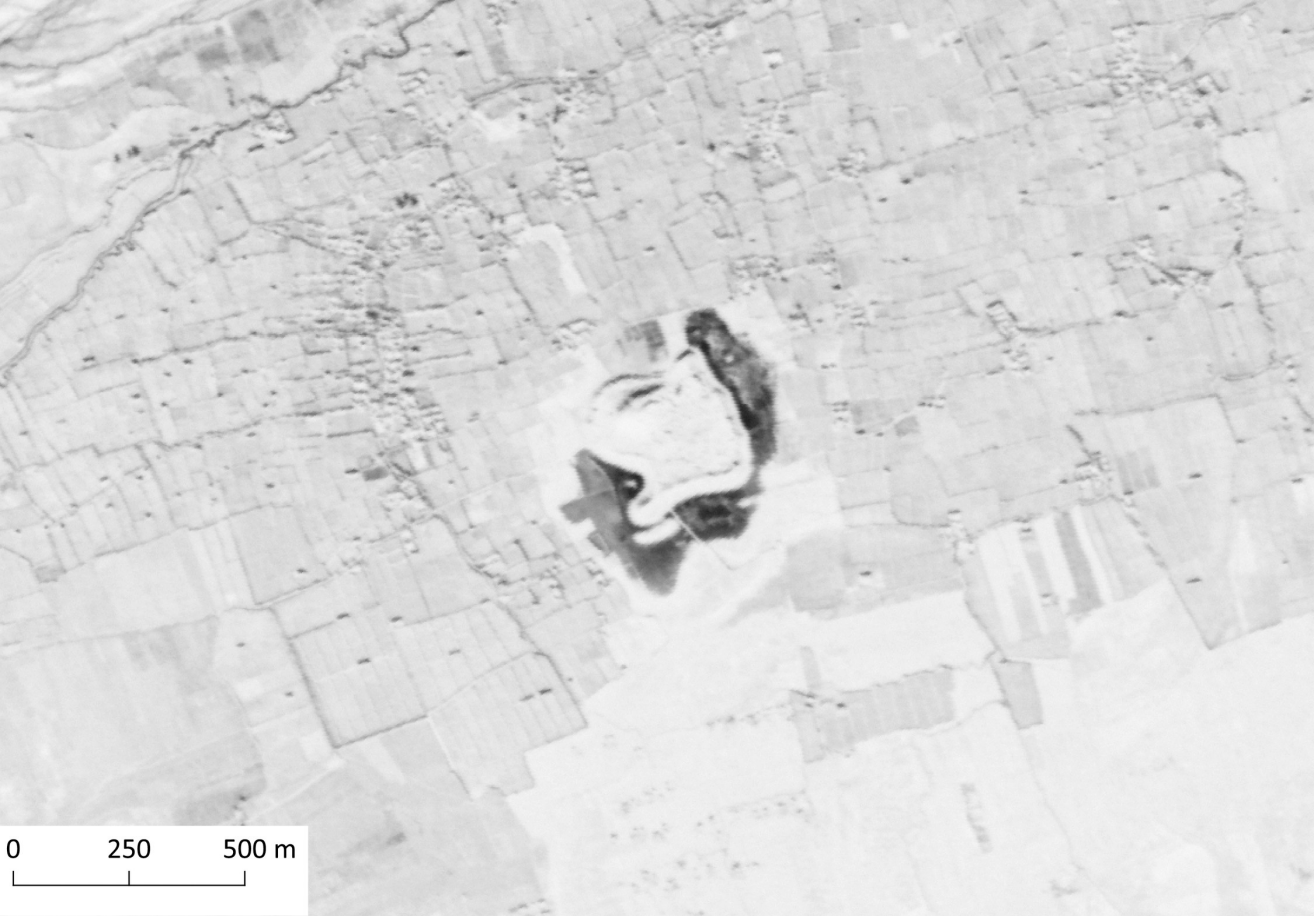
The largest settlement mound in the region, Spirwan. CORONA Satellite image courtesy of the U.S. Geological Survey (DS1108-2234DA032_a, 19.12.1969) (https://earthexplorer.usgs.gov).

Spirwan, with 9.81 ha surface area the largest mounded site in the region, is located approximately 28 km southwest of Kandahar (Fig 5). The site is situated close to a point where the Dori river joins the Arghandab. Previous surface surveys at the site documented an assemblage of Bronze Age (3^rd^ - 2^nd^ millennia BC), Indo-Parthian, Timurid and Post-Timurid periods [56, 57, 78, 79]. The mound has an irregular shape, with an inward curving bend at its southwestern side. A citadel with roundish ground plan rests on the northwestern corner of the mound. Although two gates are reportedly located on the eastern side, we were unable to confirm this observation through satellite imagery. Since the last surveys at the site were conducted in the late 1960s, it is highly likely that the soil erosion has obscured such features in the meantime. Furthermore, the site seems to be considerably damaged, with several modern structures constructed on it together with asphalt roads on and around the site. Any irreversible damage to this site would be an important loss for the prehistoric record of the region since it is probably the largest Bronze Age mound near Kandahar.

There are three other relatively larger mounds which exceed a surface area of 3 ha. These sites are located quite far from each other, with 35–40 km of distance between them (i.e. roughly a day’s travelling by foot). K686, westernmost of these sites, is located near the confluence of the Arghandab and Tarnak rivers, 10 km west of Spirwan. It is a round mound of 3.43 ha surface, with some signs of looting at its top. K445, an oval mound with 3.4 ha surface area, is located further to the east, on the upper course of the Arghandab river. It is possible that here there is a case of twin mound formation with two sites separated by a short, roughly 200 m, distance. However, due to the densely packed modern structures on each eminence, it is difficult to assess the site only by satellite imagery. While the modern structures on the larger southern mound seem a little less dense, allowing the distinct conical form of the mound to be clearly visible; the buildings on top of the northern mound cover more or less the entire surface and obscure the surface features. For this reason, the identification of a second mound on this location is somewhat uncertain. The third middle-sized mound, K168, is located quite far from the others. Unlike the alluvial flatland setting that K445 and K686 share, K168 is located within a narrow valley, along with one of the minor tributaries of the Arghandab River. The site covers 3.43 ha surface and has two round shaped cones with a circular feature on top of the more prominent southern eminence. There are some modern structures constructed on top of the site, however, not as densely packed as in the case of K445. Since none of these three sites has so far been visited during surface surveys, their dating remains to be established.

**Fig 6:**
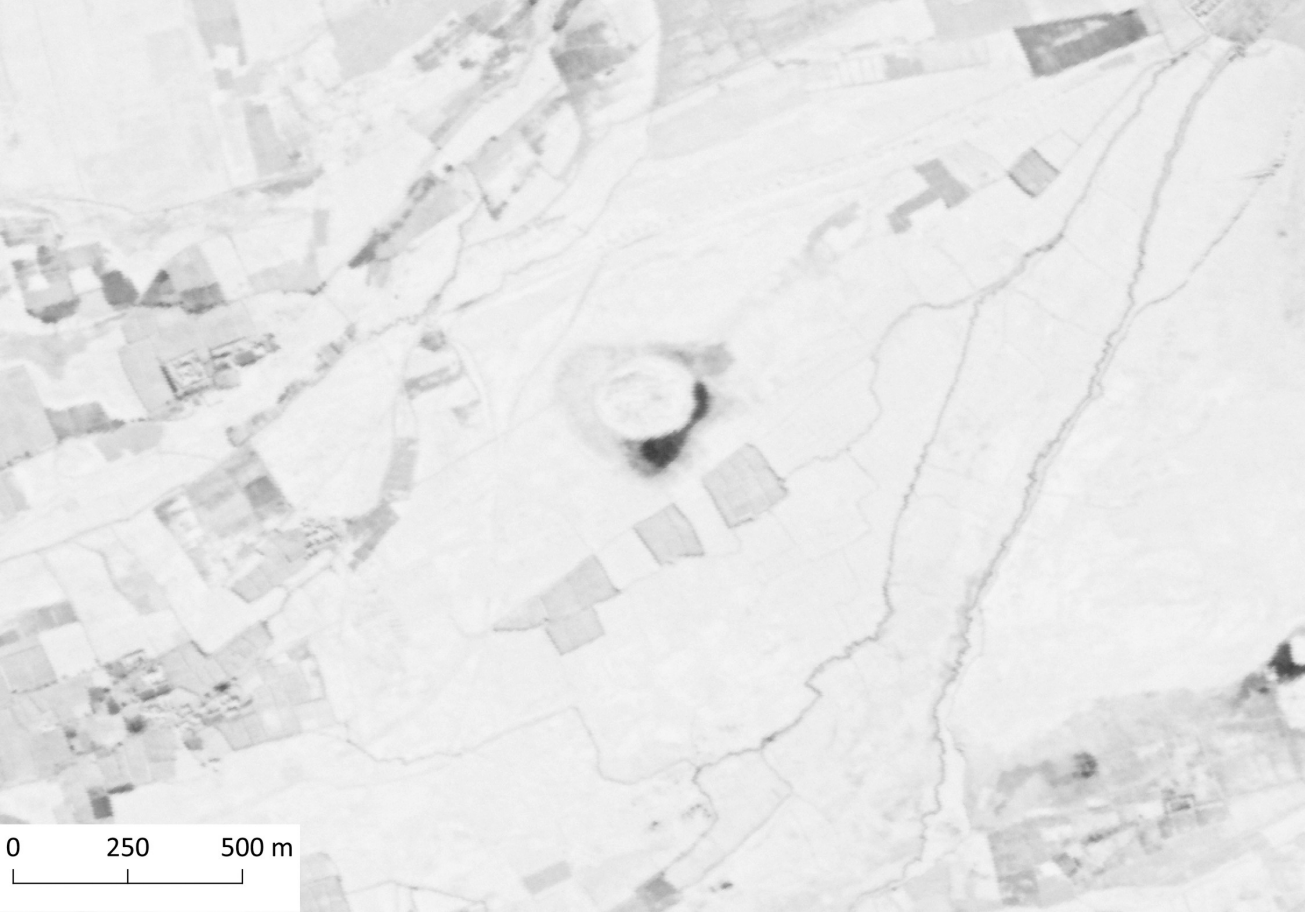
Said Qala Tepe. CORONA Satellite image courtesy of the U.S. Geological Survey (DS1108-2234DA032_a, 19.12.1969) (https://earthexplorer.usgs.gov).

There are 86 further cases where the surface area exceeds 0.35 ha. The great majority of these mounds, 67 cases, are located on the alluvial zone between the Arghandab and Tarnak. There are several substantial settlement mounds among this group with Chalcolithic and Bronze Age occupation levels that were surveyed or excavated before the 1980s, such as Said Qala Tepe [K001; 56, 57, site code: 968] (Fig 6), Deh Morasi Ghundai [K003; 56, 57, site code: 287], and Bāgh-i Pul Ghundāi [K009; 56, 57, site code: 85]. The sites that are situated outside the alluvium are more diverse in terms of their immediate environment. The parts of the Arghandab river valley north of Kandahar seem to be sparsely populated, with mounds usually having 5–10 km distance in between. Further west, within the Khākrīz valley, there is a concentration of mounded sites including Chār Sang Tepe [K158; 56, 57, site code: 180] and Mundigak [51, 54–57].

The remaining sites, where the mound formation is visible but under 0.35 ha, are also concentrated in the alluvial zone. Only ten of these sites are located north of the Arghandab River. The real difficulty with these mounds is to define exactly what kind of remains they represent. The larger ones may represent small village type settlements, hamlets or forts which are known from the Margiana and other regions of Central Asia [80–83] (K151, K456, K503, K577, K624, K661, K668). There are other cases in which several small mounds cluster together, which might be indicative of groups of burial mounds or *kurgan* cemeteries (K566, K659). The square or rectangular footprints in some cases suggest that they might be remains of shrines, stupas, or other architectural remains that are completely eroded (K328, K593, K655, K656). These are, however, limited cases and most of the mounds under 0.35 ha are completely devoid of such characteristic details.

### Architecture and ruins

28 locations with clearly recognizable architectural features in varying states of preservation were spotted on the satellite imagery. Only one of these sites, Chālgūr [K008; 56, 57, site code: 167], where a single rectangular structure with four towers in each corner is present, was previously known and it is dated to the Late Antique and Early/High Medieval periods [56, 57]. The other recorded structures are quite diverse in terms of their shape and size. While most of them are under 0.2 ha, larger and more elaborate examples also occur. There are two cases where several structures cluster together (K303, K361), which possibly represent building complexes of administrative, religious or military purposes. Beyond these two cases, though, most other structures seem to be small, singular structures of undetermined function. In any case, a surface survey would be required to fully establish their architectural characteristics as well as their dating.

### Stupas

**Fig 7:**
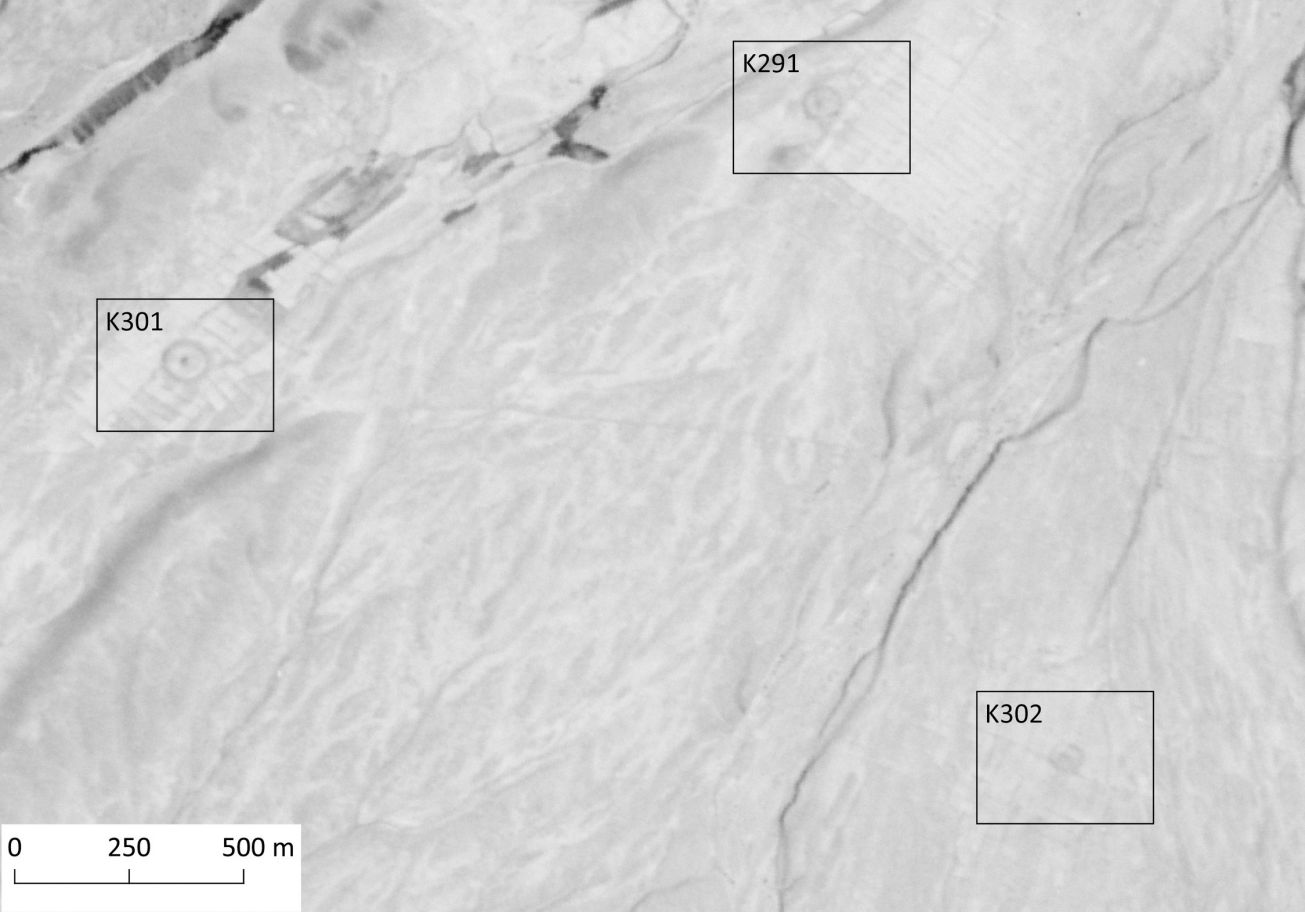
Group of stupas: K291, K301, and K302. CORONA Satellite image courtesy of the U.S. Geological Survey (DS1021-2119DF015_b, 05.26.1965) (https://earthexplorer.usgs.gov).

As a central element of Buddhist religious architecture, the stupa is a stylized form of burial mound with a conical shape, in which physical remains of Buddhist nuns and monks, or other relics from Buddha and his disciples are interred [84, 85]. Stupas are often built within monastic complexes, cities, or places of pilgrimage, and they act as places of worship and meditation. Stupas can be recognized in the satellite imagery as small, mound-like conical features surrounded by one or more rings. These rings consist of earthworks and ditches, and in some cases, several rings may be surrounding the stupa in a concentric layout. Although the rings vary in size, most of them are constructed as near-perfect circles, and therefore they can be quite distinctive when seen from above (Fig 7).

Pre-Islamic religious and ritual sites (stupas, fire temples) were at times overbuilt subsequently by Islamic ritual structures like shrines and mosques [86–88]. Indeed, we were able to identify 14 features located within the study area, which are most likely remains of stupas. These features consist of circular or square mounds, with one or two concentric rings around them (K356 is the only feature with two concentric rings around the main structure). There are also some cases where the rings are interrupted by a single causeway. The diameters of the outermost rings range between approximately 25 to 100 m. While most of the stupas were free of any clear signs of disturbances, four sites (K291, K293, K301 and K583) seem to have been illicitly excavated, as there are many visible pits on and around the main structures.

The stupas in the Kandahar region are situated on various types of terrain without a selective pattern; examples have been recorded on hillsides, river banks or flatlands. Except for one case where four stupas cluster together (K291, K293, K301 and K302), they are distributed as singular features across the landscape. It should also be noted that a part of the smaller mounds discussed above, those without conspicuous features such as rings, could also represent the remains of stupas, although this must remain uncertain given the lacking characteristic details. For this reason, the actual number of stupas in the region can be significantly higher than the ones we were able to identify during this study.

### Fortified sites and fortifications

**Fig 8:**
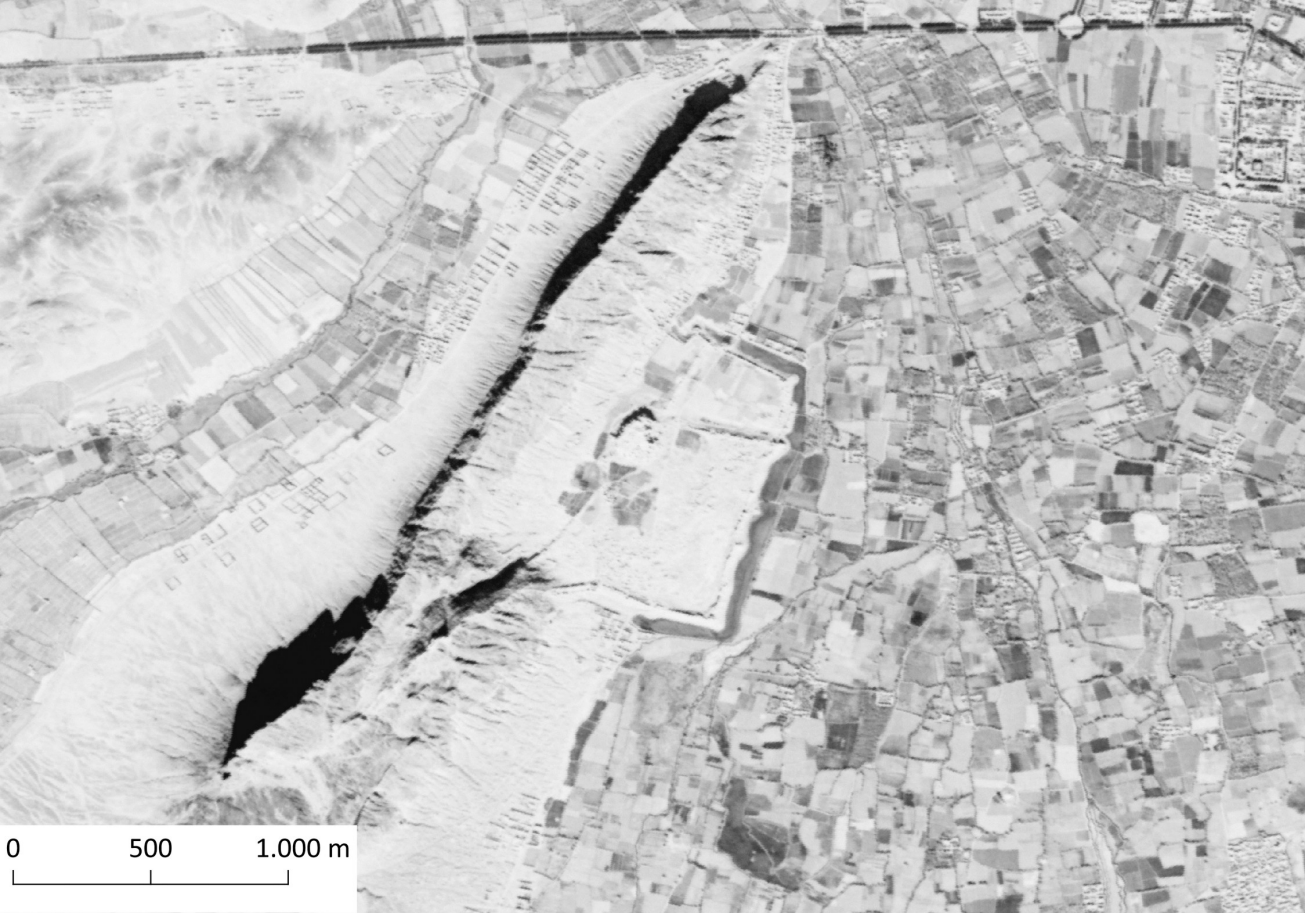
The old city of Kandahar. CORONA Satellite image courtesy of the U.S. Geological Survey (DS1108-2234DA032_a, 19.12.1969) (https://earthexplorer.usgs.gov).

Defensive structures such as mudbrick fortresses and fortifications are quite common features of the historical landscape in Afghanistan. Five such features have been previously identified in the region through conventional surveys: the fortifications of the old city of Kandahar [K007; 56, 57, site code: 522] (Fig 8), Lalkhān Qala [K004; 56, 57, site code: 672], Sang-i Sar [K013; 56, 57, site code: 988], Sabz Qala [K016; 56, 57, site code: 962], and Kushk-i Nakhud [K017; 56, 57, site code: 659]. We were able to locate another fortress (K713), situated roughly 20 km south of the Kandahar city centre, overlooking the Arghandab river valley. The fortress K713 is constructed on relatively flat terrain, on a roughly square plan and it covers an area of approximately 2.32 ha. The structure shows discernible signs of erosion on all sides, and there are visible looting pits especially on the southern side.

**Fig 9:**
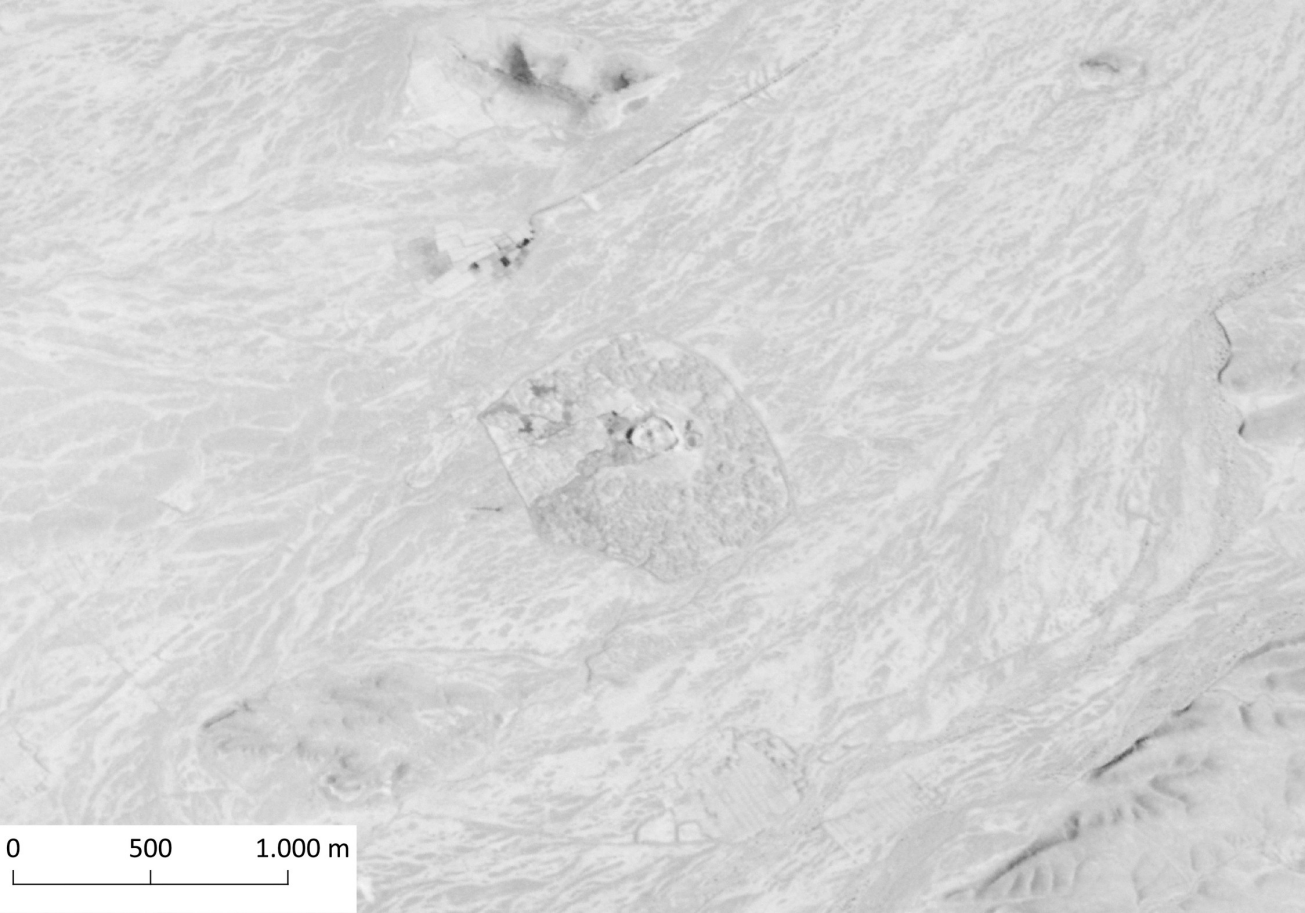
K311, the ruins of a fortified town with a citadel at its centre. CORONA Satellite image courtesy of the U.S. Geological Survey (DS1021-2119DF015_b, 05.26.1965) (https://earthexplorer.usgs.gov).

The largest and probably the best-preserved settlement site encountered during the survey was K311 (Fig 9). A walled town with a citadel at its centre, it covers a surface of 80 ha in the westernmost parts of our study area, at midway between the villages of Amanat Baba and Garmabak Shamali. The fortifications around the town have a round ground plan, except for the eastern, western, southeastern and southern sides where the walls are built straight and join in angles. Remains of the structures are clearly discernible within the walls, especially in the southern half of the settlement. The buildings seem to have rectangular plans, in some cases with shared walls and possibly multiple rooms. While the site appears quite well-preserved, there are certain parts with obvious damage. The immediate surroundings of the citadel are completely devoid of any surface features, possibly due to erosion. The western side of the citadel, as well as the northwestern corner of the fortified area, is cultivated. Dirt roads can be seen cutting across the site from the northwest towards east and south. Nevertheless, a surface survey at the site could document and register a large number of structures, and possibly establish a relative dating for the site without the necessity of excavations.

### Qanats/Karez

*Qanats* (or *Karez* in Pashto) are sloping underground channels with a series of vertical access shafts that provide freshwater for irrigation and stock breeding as well as drinking water [89]. The origins of the technology are traced back to Iron Age Iran [90, 91]. They are not only an essential irrigation technique but also cultural heritage that deserves to be protected and preserved [92]. *Qanats* rely on gravity to deliver water from higher elevations such as hillsides or mountain slopes, down to the settlements and cultivated areas that are located on lower elevations [93]. The lack of need for fuel makes *qanats* a completely carbon-neutral and sustainable water management strategy [94]. Around 6700 are known across Afghanistan [95], and recently, a large number of *qanats* have been documented through satellite imagery in southern and central Afghanistan, including also the northern part of the Registan Desert [42, 96].

**Fig 10:**
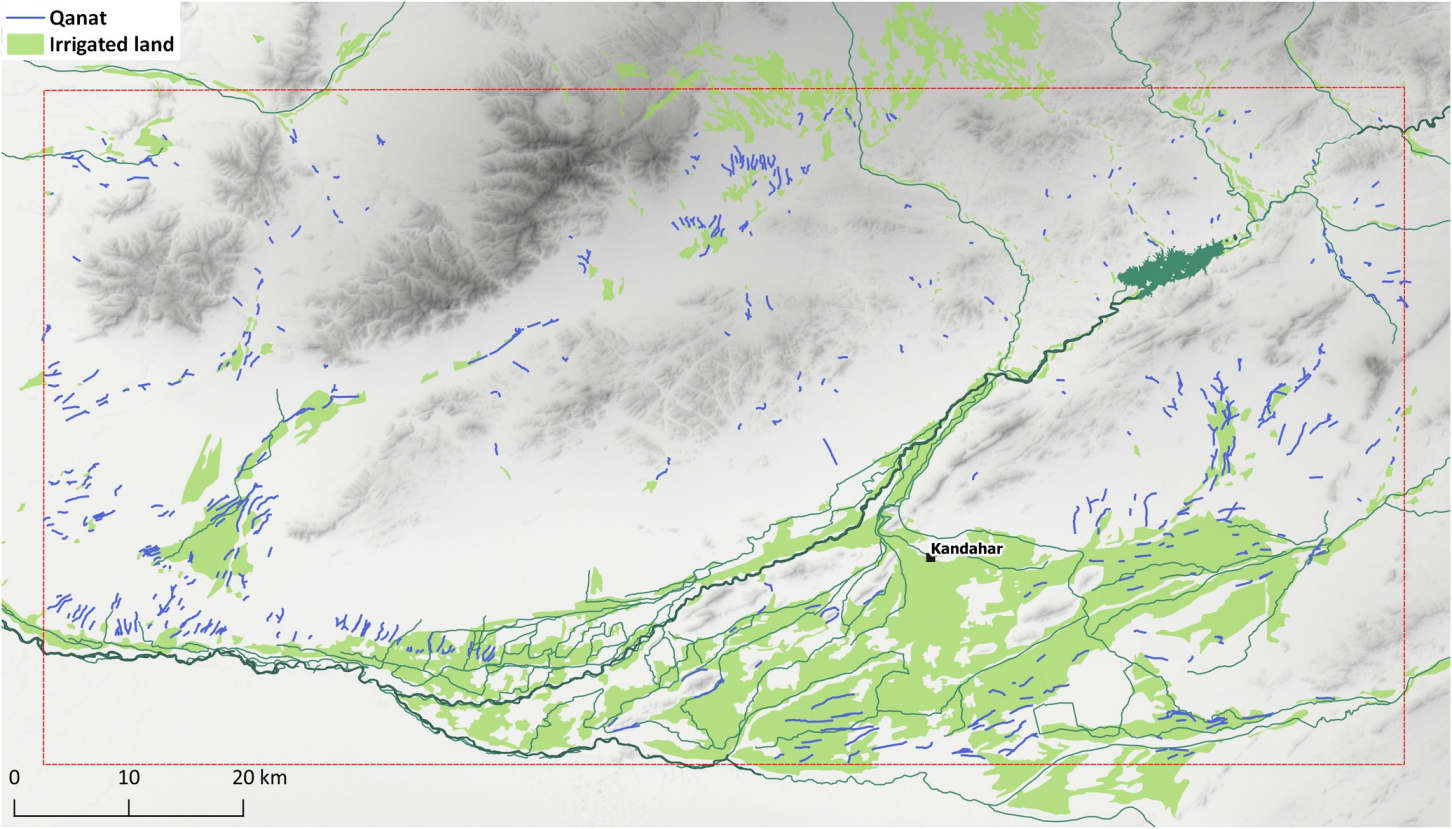
Map showing the distribution of *qanats*. DEM based on Shuttle Radar Topography Mission (SRTM) 1 Arc-Second Global courtesy of the U.S. Geological Survey (doi:10.5066/F7PR7TFT) (https://earthexplorer.usgs.gov). The map was created by the authors in QGIS.

**Fig 11:**
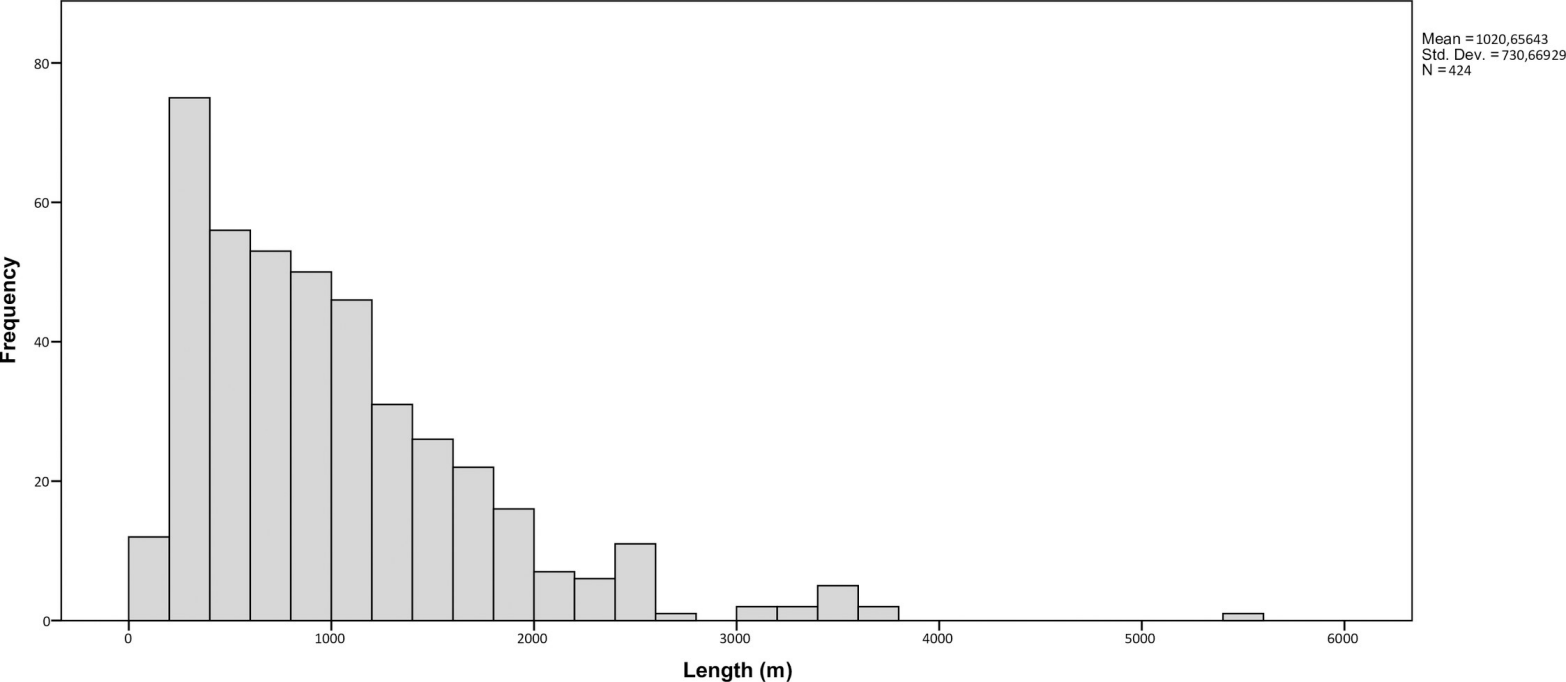
Histogram showing the length range of *qanats*.

**Fig 12:**
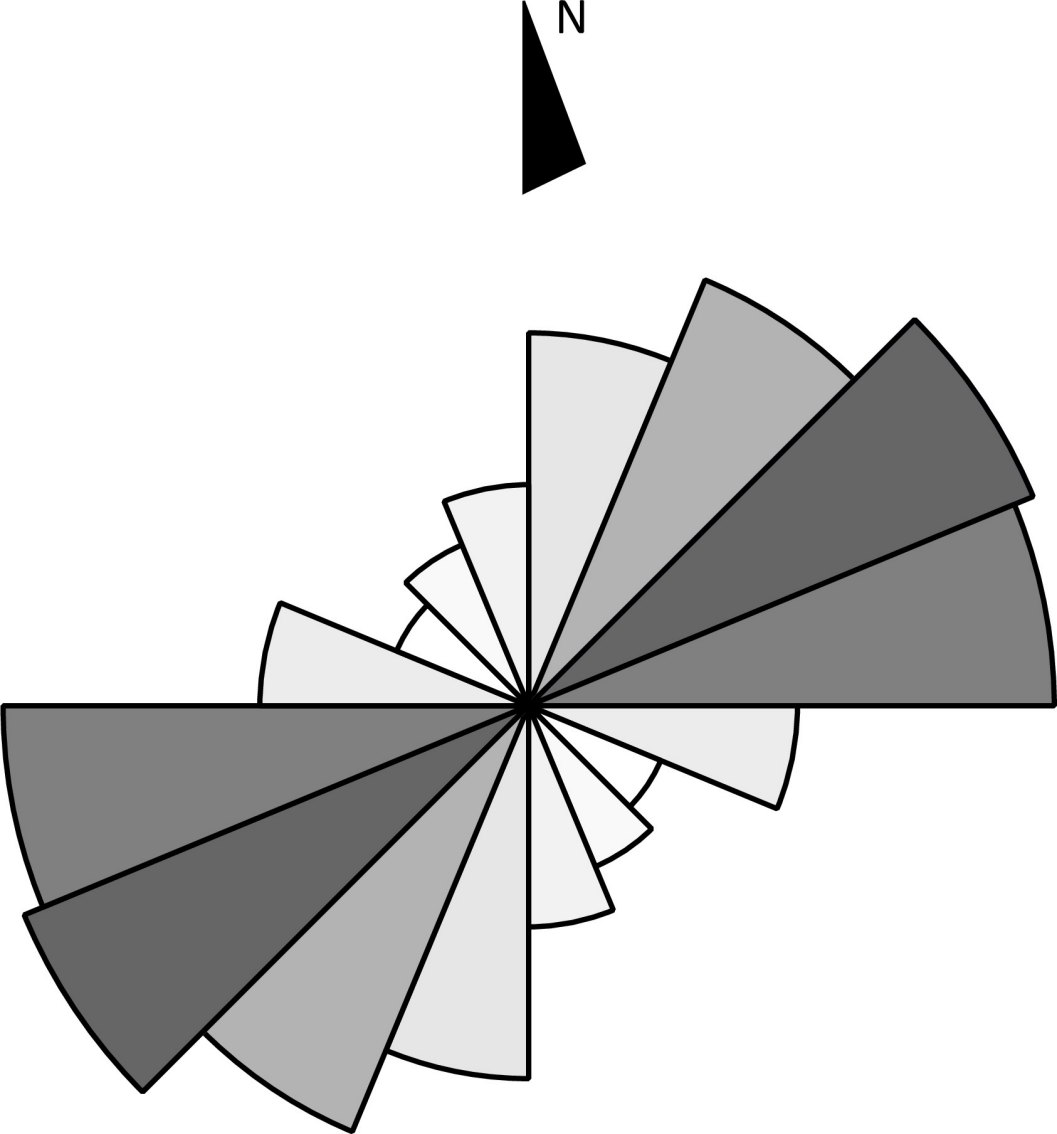
Rose-chart showing the directional tendency of *qanats*.

*Qanats* are the most abundant type of feature that we have come across during this study. All together 582 examples of these traditional water management structures were spotted. Except for their tendency to be located in the areas where the terrain allows the cultivation of crops, *qanats* do not reveal any other obvious spatial patterns in their distribution (Fig 10). The most apparent concentration of *qanats* is located in the southern and eastern parts of the alluvial zone around Kandahar, the Khākrīz valley, Ghorakh valley, and along the Arghandab River. Lengthwise, they have considerable variation; while the shortest *qanat* in the region is just 80 m long, the longest one spans 5.5 km. Still, more than half of the *qanats* in Kandahar cover a distance under 1 km, and except a single case (K728–5.5 km long), they are all under 4 km (Fig 11). Most *qanats* in the region tend to have northeast-southwest alignment, whereas *qanats* that stretch from east to west are also common. *Qanats* that cross from north to south are much less frequent (Fig 12).

## Discussion

**Fig 13:**
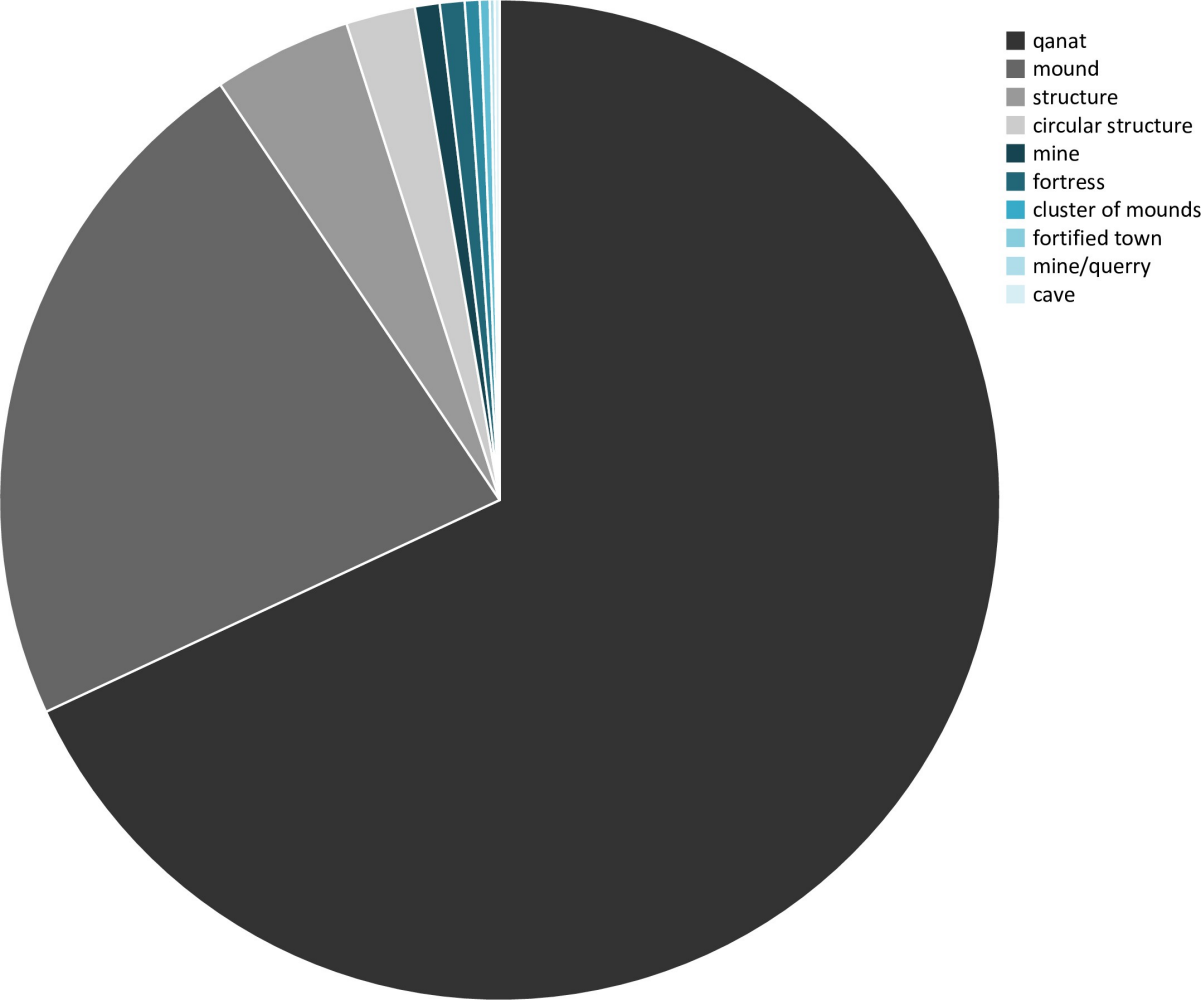
Pie-chart showing the ratio of site-types.

**Fig 14:**
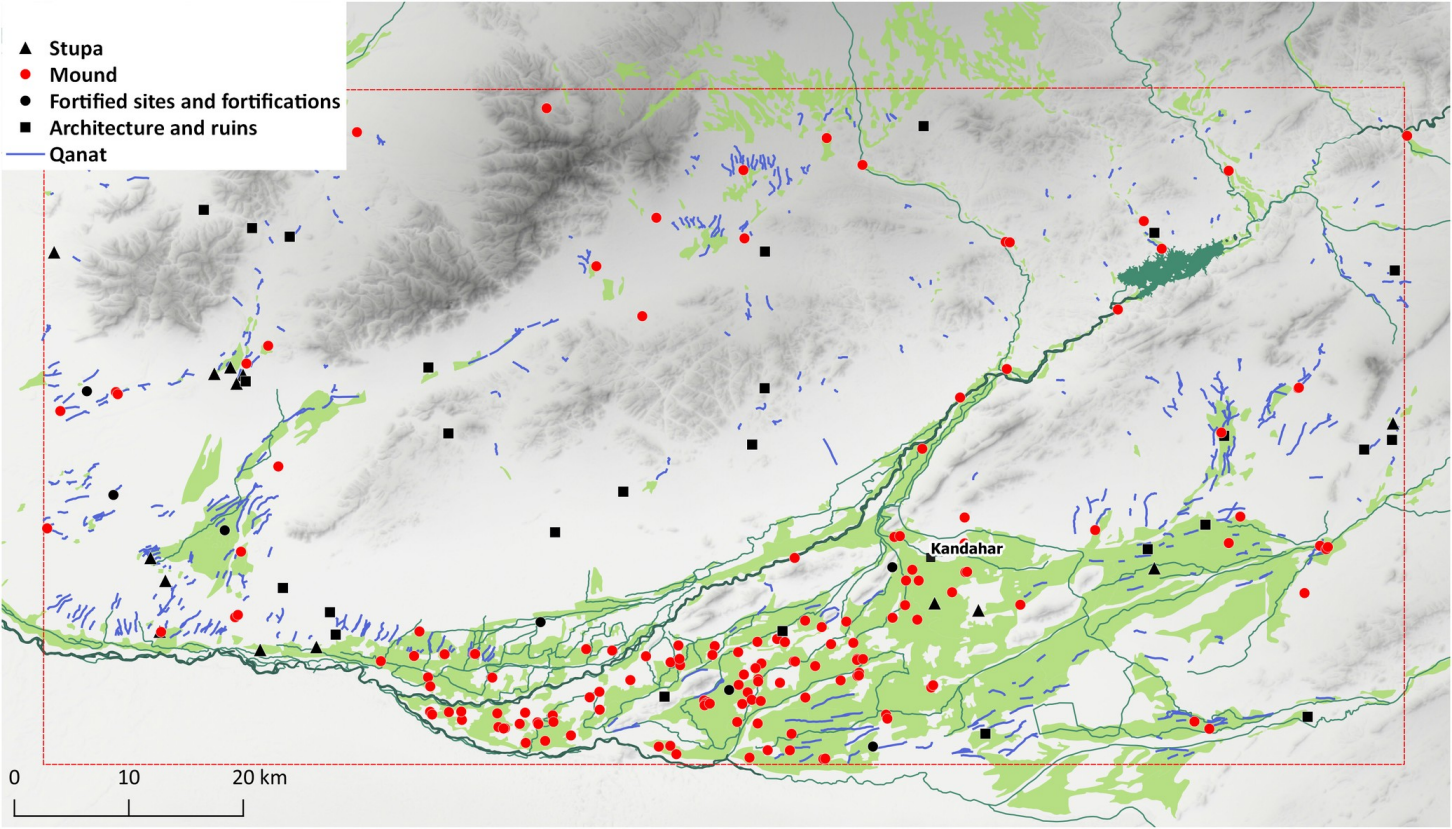
Map showing the distribution of all sites. DEM based on Shuttle Radar Topography Mission (SRTM) 1 Arc-Second Global courtesy of the U.S. Geological Survey (doi:10.5066/F7PR7TFT) (https://earthexplorer.usgs.gov). The map was created by the authors in QGIS.

Previous research established that remote sensing-based archaeological surveys offer valuable data in those regions that are inaccessible to archaeologists for a multitude of reasons including conflict and political turmoil. With this study, we have attempted a remote-sensing survey of the Kandahar region by using only publicly available and open-source resources. The method is particularly suitable for identification of sites that have conspicuous terrain features such as mounds, sites that consist partly of open remains of architectural elements, as well as long linear structures such as *qanats* (Figs 13 and 14). It can, however, be assumed that these reflect only a part of the region’s diverse heritage.

The most common features that we have encountered in Kandahar were the *qanats*, which are an essential component of the traditional water supply network in the region. *Qanats* are thought to be introduced in Afghanistan during the Achaemenid Period, but their roots are traced back to Bronze Age Iran [97]. Their distribution area includes the highlands to the south and west of the Hindu Kush Mountains but they are absent on the northern side of the Hindu Kush [89]. *Qanats* are used in dryland regions that are often lacking permanent and reliable watercourses, and therefore they are vital to sustaining human life and agricultural production [93]. The construction and maintenance of *qanats* rely on specialist skill and knowledge, and the distribution of water through networks of *qanats* often have implications on land and water rights as well as the organization of agricultural labour. *Qanats* are not only a technological solution to food production in arid environmental conditions but also a cultural mechanism that is entangled with the social and economic organization of the communities relying on them. As such, they represent both the tangible and intangible aspects of heritage with the traditional knowledge and skillsets associated with their construction as well as social and cultural practices that regulate their use. Therefore, the preservation of *qanats* as heritage depends on protecting the embedded knowledge through promoting their sustained use beyond mere conservation of the existing *qanats*. Abrupt changes in water management strategies in regions where *qanats* and other traditional technologies have been relied upon since centuries might even result in socio-political issues and conflict [42, 98, 99].

Heritage concerns in Afghanistan are manifold and they are deeply intertwined with the course and events of the conflict that has been raging for the past four decades [2, 3, 100]. The looting of cultural heritage and the subsequent illicit trade of heritage assets as a means to finance armed conflicts around the world has been investigated in detail [101, 102], and the recent assessments have shown that Afghanistan remains to be a primary locus for such activities at present [6, 103]. Once taken out of Afghanistan, and into neighbouring transit countries such as Pakistan and Iran, these looted objects find their way to their intended markets, among which the U.S., the U.K. and Germany seem to be prominent [102]. It is, then, imperative that the heritage specialists and other responsible parties in Afghanistan have the necessary tools and data at their disposal to monitor and prevent these destructive acts which constitute the first step in this transnational criminal enterprise.

Satellite imagery can be used to assess and monitor the damage at archaeological sites which result from illicit excavations and looting. Looting pits form distinctive features in the landscape due to their characteristic shape, shallow depth, and the mounds of debris accumulating around them. In some cases, looting pits form large clusters, and thus, make their identification from space easier [103–105]. The imagery used in this study also provides enough resolution to assess the presence or absence of looting pits on the archaeological sites. Indeed, a large number of sites recorded during this survey show signs of damage related to various factors, with looting pits as the most prominent among them. Looting pits were observed in more than three-quarters of all non-*qanat* sites during this survey (159 out of 202 cases). Mound type sites are also severely affected by illicit excavations, as nearly half of them are noticeably marked with clusters of looting pits (67 out of 138 cases). Other than the looting pits, primary causes of damage in archaeological sites seem to be the soil erosion and agricultural production. Terracing around the archaeological sites to increase the size of cultivated fields, as well as modern construction activities also result in considerable damage in many cases.

Although it is not within the scope of this paper, a comparison of the site data presented here with legacy satellite imagery (such as CORONA) may help expose the patterns and processes associated with the destruction of cultural heritage in Afghanistan. Archives consisting of satellite imagery collected over a long-time span (decades in the case of CORONA) provide information on the status of heritage before the expansion of industrialized agriculture and the growth of urban areas in many parts of the world [106]. Both factors have a considerable impact on the preservation of cultural heritage, therefore, it is crucial that they are framed as long-term processes and their impact is studied accordingly. A recent study by Hammer and colleagues find an overall upward trend in the damage related to agriculture, looting, land development, and military activity between 2000 to 2017 [6]. Likewise, a study in Syria that focuses on the impact of Syrian Civil War on cultural heritage reveals the dramatic intensification of looting as a result of worsening conflict conditions [39, 107]. The WMS data sources employed in this study, however, do not provide the full range of information (such as the date of acquisition), and sorting capabilities needed to conduct a diachronic evaluation of the features that have been located.

Beyond their value as part of the region’s rich cultural heritage, the mounds in the Kandahar region may help resolve some of the outstanding questions in archaeological research. Previous studies and fieldwork provided evidence that the region was involved in the networks that were responsible for the procurement and distribution of resources such as *lapis lazuli*, copper, and tin which were in high demand in Mesopotamia during the Chalcolithic period and the Bronze Age [58–60, 95]. The settlement mounds in Kandahar may hold important clues to the organization of these networks, as these sites were situated directly on the routes that would connect the Hindu Kush highlands to the Iranian Plateau towards the west. As such, future excavations at these sites may reveal some of the local processes behind this supra-regional phenomenon, and help us understand to which extent the socio-economic and political processes in Central Asia were intertwined with those in Iran and Mesopotamia during the Bronze Age. Moreover, the stratified deposits within these mounds may have occupational layers predating the Chalcolithic period and may reveal how sedentism and farming began in the region.

Unlike the areas north of the Amu Darya River where towns of urban proportions, such as Dzarkutan in Uzbekistan or Gonur Depe further west in the Karakum Desert of Turkmenistan, appear already during the Early Bronze Age [82], there is presently no evidence to suggest that urban centres developed in the Kandahar region before the Achaemenid Period. It seems likely that Kandahar was the first urban centre to flourish in the region and kept its status as a regional centre until today. However, the presence of K311 with an 80-ha fortified area shows that there was at least one more major town nearby Kandahar at some point in time. A future visit to this site to establish its dating would help contextualise it better within the region’s history. In this respect, the major short-coming of satellite-based detection of archaeological sites is that the method rarely delivers any information on the temporal aspect of the sites with some exceptions such as architectural elements that may contain stylistic traits which permit a relative dating. Therefore, it is not possible to determine the time range during which these mound sites were occupied and to begin exploring some of the questions mentioned above in more detail. Nevertheless, the alluvial plains between the Arghandab and Dori rivers are densely occupied by settlement mounds, some of which may have occupational histories extending back to the 4^th^ Millennium BC, possibly even earlier. Surface surveys at the sites presented in this paper are necessary to establish their dating and to put the site distribution data in a diachronic perspective. The on-going communication with colleagues from the Institute of Archaeology of Afghanistan has already contributed to this study by increasing our ability to locate and define certain elements, especially the architectural remains and stupas. Continued cooperation in these areas will certainly help us address these points.

## Conclusions

With this study, we were able to record a large and diverse group of sites that are of archaeological interest in a region that was previously lacking sufficient data even for a basic understanding of the archaeological and heritage potential. The results are significant since they clearly demonstrate how great a gap there is between what we currently know about the past of Afghanistan and what we may actually find out by targeted, intensive research. The new data presented here may benefit future survey projects in the region by providing a road map for potential points of interest. The results also demonstrate that the cultural heritage situation in Afghanistan is at a tipping point. Many sites in the region are threatened by a multiplicity of factors such as looting, urban development, soil erosion, and agricultural practices. Nonetheless, the location data that we provide in the open-access appendix may assist the local authorities and specialists in Afghanistan in their efforts to monitor and preserve these sites (S1 Appendix). The ongoing destruction of sites in such an understudied region, however, has implications beyond the sphere of cultural heritage management; as sites of critical value like Spirwan are lost, it will be impossible to fully assess and reconstruct the past cultural and social processes in the region.

## Supporting information

S1 Appendix List of remotely surveyed archaeological/heritage sites near Kandahar and their locations (WGS84). (PDF)
