# Supplementary material for: A remote sensing-based survey of archaeological/heritage sites near Kandahar, Afghanistan through publicly available satellite imagery
Source: PLoS One. 2021 Nov 2;16(11):e0259228. doi: 10.1371/journal.pone.0259228 (PMC8562850; doi:10.1371/journal.pone.0259228)
Supplement: S1 Appendix — (PDF) [file pone.0259228.s001.pdf]

id.	Name	Long.	Lat.	Type	Area (ha)	Length (m)	Width (m)
K001	Said Qala Tepe	65.5795897	31.5520628	mound	2.91	213	185
K002	Spirwan	65.4190736	31.4974276	mound	9.81	318	332
K003	Deh Morasi Ghundai	65.4965201	31.5286776	mound	0.87	140	72
K004	Lalkhān Qala	65.5097988	31.4883405	fortress	1.44	120	119
K005	Panjwāyi	65.4632091	31.5292420	mound	1.22	145	120
K006	Salawāt	65.5539153	31.5351690	mound	0.84	121	88
K007	Kandahar	65.6597054	31.6010976	fortified town	64.47	1114	592
K008	Chalgūr	65.5588446	31.5424404	structure	0.35	76	41
K009	Bāgh-i Pul Ghundāi	65.5699884	31.6096457	mound	2.04	199	133
K010	Shamshir Ghar	65.5000015	31.5833292	cave			
K011	Zala Khan	65.5948147	31.5461226	mound	0.41	69	67
K012	Nādir Tepe	65.6239213	31.5316052	mound	0.38	71	61
K013	Sang-i Sar	65.3365794	31.5506968	fortress	1.72	298	46
K014	Zakird	65.7566070	31.5433705				
K015	Shahr-i Gai	65.6181888	31.6804528				
K016	Sabz Qala	64.9442698	31.6674827	fortress	0.07	34	30
K017	Kushk-i Nakhud	65.0465087	31.6350109	fortress	1.5	121	106
K018	Arūkh South	65.5023900	31.8841485				
K019	Mundigak	65.5238818	31.9031595	mound			
K020	Bād-i Sah Ghundāi	65.8516189	31.6010468				
K021	Sāhibzāda Qal'acha	65.8931461	31.6036130				
K102		65.3421661	32.0227020	mound	0.05	28	26
K103		65.0303573	32.0162127	structure	0.29	62	44
K104		66.0931163	31.7091905	structure	0.07	26	24
K105		65.5768593	32.0158826	qanat		1235	
K106		65.5997769	32.0173238	qanat		1374	
K107		65.6201907	32.0163395	qanat		1067	
K108		65.6567657	32.0123547	qanat		644	
K109		65.6614912	32.0144286	qanat		1730	
K110		65.9598686	32.0195319	qanat		527	
K111		65.9739564	32.0103252	qanat		238	
K112		65.9859037	32.0195171	qanat		408	
K113		66.1076315	32.0120176	qanat		210	
K114		65.1382717	32.0064699	structure	0.07	30	23
K115		65.1679805	32.0007592	mound	0.62	76	67
K117		65.6254791	32.0062773	qanat		445	
K118		65.6884744	32.0063366	structure	0.07	30	26
K119		65.9492250	32.0033368	qanat		215	
K120		64.9352945	31.9923303	qanat		595	
K121		64.9911347	31.9963225	qanat		455	
K122		65.1091998	31.9945301	qanat		578	
K123		65.1110219	31.9987964	qanat		815	
K124		65.1885179	31.9943276	qanat		733	
K125		65.4798110	31.9987106	qanat		315	
K126		65.5994482	31.9953197	mound	1.5	130	116
K127		65.9171060	31.9922915	qanat		276	
K128		65.9257610	31.9969411	qanat		126	

K129		66.1325514	31.9973983	mound	1.42	136	117
K130		64.9021890	31.9810212	qanat		425	
K131		64.9453825	31.9752092	qanat		780	
K132		64.9756046	31.9766752	qanat		2013	
K133		65.0020878	31.9714111	qanat		356	
K134		65.5170333	31.9852635	qanat		1692	
K135		65.9891334	31.9808139	qanat		347	
K136		65.4781933	31.9766011	qanat		540	
K137		65.5069124	31.9674649	qanat		1450	
K138		65.5129095	31.9724254	qanat		1958	
K139		65.5167372	31.9750982	qanat		474	
K141		65.5229489	31.9744392	qanat		849	
K142		65.5267989	31.9748835	qanat		1090	
K143		65.5312930	31.9744615	qanat		1351	
K144		65.5354761	31.9734397	qanat		1268	
K145		65.5384820	31.9738247	qanat		671	
K146		65.5407624	31.9713445	qanat		615	
K147		65.5457155	31.9721071	qanat		482	
K148		65.5422135	31.9730695	qanat		845	
K149		65.5506464	31.9740098	qanat		1075	
K150		65.6232883	31.9747317	mine			
K151		65.6323060	31.9706670	mound	0.3	45	32
K152		66.0467161	31.9739247	qanat		317	
K153		64.9084674	31.9694010	qanat		357	
K154		64.9175814	31.9687569	qanat		1336	
K155		65.1280848	31.9688975	qanat		731	
K156		65.4717693	31.9636902	qanat		687	
K157		65.4773698	31.9690736	qanat		247	
K158	Chār Sang Tepe	65.5229440	31.9658620	mound	0.42	86	74
K160		65.5513218	31.9621305	qanat		522	
K161		65.5555995	31.9610907	qanat		1259	
K162		65.5633125	31.9610775	qanat		1324	
K163		65.5786597	31.9638021	qanat		733	
K164		65.5817726	31.9665135	qanat		351	
K165		65.8456290	31.9602944	qanat		81	
K166		65.8611999	31.9638153	qanat		834	
K167		65.8687024	31.9673559	qanat		152	
K168		65.9686629	31.9652829	mound	3.65	346	180
K169		64.9362841	31.9518113	qanat		568	
K171		64.9663598	31.9590835	qanat		1881	
K172		65.1490185	31.9588794	qanat		288	
K173		65.5331381	31.9540160	qanat		391	
K174		65.5473139	31.9541016	qanat		1042	
K175		65.8817593	31.9533579	qanat		218	
K176		65.9382581	31.9583200	qanat		100	
K177		66.1035164	31.9527195	qanat		656	
K178		64.8876107	31.9441106	qanat		200	
K179		65.5480688	31.9445430	qanat		259	
K180		65.8015906	31.9452976	qanat		637	

K181		65.8228376	31.9408070	qanat		412	
K182		65.1415987	31.9397286	qanat		294	
K183		65.1773325	31.9305123	qanat		225	
K184		65.6742245	31.9332860	qanat		295	
K185		66.0860612	31.9379312	qanat		264	
K186		66.1058105	31.9331011	qanat		600	
K187		65.0272522	31.9293806	structure	0.11	46	30
K188		65.1472942	31.9271024	qanat		762	
K189		65.4429796	31.9221095	mound	1.92	201	105
K190		65.4663571	31.9188809	qanat		789	
K191		65.4695969	31.9206043	qanat		1134	
K192		65.4761874	31.9191545	qanat		703	
K193		65.4969649	31.9209963	qanat		1667	
K194		65.5650372	31.9275573	qanat		89	
K195		65.8791997	31.9295285	qanat		815	
K196		65.9133949	31.9262887	qanat		301	
K197		65.9589294	31.9224868	qanat		417	
K198		65.9529232	31.9185665	qanat		419	
K199		65.0715921	31.9126417	structure	0.18	31	30
K200		65.0853057	31.9168652	qanat		529	
K201		65.1134725	31.9104153	qanat		1226	
K202		65.1528085	31.9182188	qanat		214	
K203		65.4604101	31.9143947	qanat		803	
K204		65.4848490	31.9149347	qanat		806	
K205		65.4975862	31.9118059	qanat		1188	
K206		65.5005153	31.9102082	qanat		722	
K207		65.5031781	31.9107259	qanat		1222	
K208		65.5254350	31.9162365	qanat		989	
K209		65.8907017	31.9190547	mound	1.8	190	110
K210		66.1239289	31.9119908	qanat		333	
K211		65.0895588	31.9007773	qanat		444	
K212		65.1061941	31.9047124	structure	0.33	66	50
K214		65.3646073	31.9044313	qanat		385	
K215		65.5296955	31.9077007	qanat		289	
K216		65.7638103	31.8998971	mound	0.19	32	30
K217		65.7675975	31.8995420	mound	0.17	45	41
K218		65.7993591	31.9085957	qanat		457	
K219		65.8971813	31.8999341	qanat		977	
K220		65.8935051	31.9073382	qanat		177	
K221		65.9004210	31.9082628	structure	0.42	66	58
K222		65.9174336	31.9080187	qanat		225	
K223		66.0564353	31.9097533	qanat		527	
K224		64.8897968	31.8904647	circular structure	0.24	53	52
K225		65.4895467	31.8923347	qanat		1436	
K226		65.5427108	31.8910412	structure	0.45	99	42
K227		65.7319966	31.8990458	qanat		164	
K228		65.9071728	31.8936355	mound	2.03	205	104
K229		66.0605674	31.8929112	qanat		712	
K230		64.9095292	31.8848086	qanat		638	

K231		65.3791807	31.8825213	qanat		1261	
K232		66.0463866	31.8830830	qanat		314	
K233		66.0722897	31.8844871	qanat		447	
K234		66.0963970	31.8802672	qanat		521	
K235		65.0774190	31.8734238	qanat		587	
K236		65.3878348	31.8776067	mound	1.34	192	77
K237		65.3767197	31.8776659	qanat		1153	
K238		65.8555167	31.8793435	qanat		373	
K239		66.0728070	31.8734386	qanat		258	
K240		66.1206077	31.8719457	qanat		1001	
K241		66.1212284	31.8736825	structure	0.24	65	62
K242		65.0793109	31.8616658	qanat		775	
K243		65.6782635	31.8657194	qanat		361	
K244		65.6849813	31.8595337	qanat		275	
K245		65.6949656	31.8634653	qanat		353	
K246		65.8473171	31.8662145	mine			
K247		66.0773632	31.8675670	qanat		818	
K248		66.1152608	31.8633840	qanat		916	
K249		66.1278908	31.8630810	qanat		882	
K250		66.1025741	31.8499867	qanat		1085	
K251		65.0573720	31.8463143	qanat		1034	
K252		65.0777038	31.8399481	qanat		1089	
K253		65.5270625	31.8446599	qanat		807	
K254		65.5452968	31.8443645	qanat		1446	
K255		65.7482523	31.8467131	qanat		820	
K256		65.8483674	31.8475550	qanat		196	
K257		66.1264868	31.8467389	qanat		396	
K258		65.4300237	31.8317252	mound	1.05	120	102
K259		65.5261138	31.8328107	qanat		824	
K260		65.5283069	31.8326778	qanat		821	
K261		65.8668985	31.8378153	mound	1.63	154	142
K262		64.8832080	31.8177747	qanat		712	
K263		65.0973269	31.8203444	qanat		731	
K264		65.3339824	31.8249964	qanat		1149	
K265		65.3447929	31.8256905	qanat		1399	
K266		65.7647088	31.8289026	qanat		267	
K267		65.0647110	31.8155151	qanat		595	
K268		65.0698947	31.8159212	qanat		919	
K269		65.3155035	31.8164344	qanat		1104	
K270		65.5088323	31.8147545	qanat		510	
K271		65.7093755	31.8131263	qanat		158	
K272		65.0863850	31.8045005	mound	0.4	80	64
K273		65.2999653	31.8026037	qanat		1856	
K274		65.6678542	31.8000501	qanat		742	
K275		66.0525741	31.8027772	qanat		433	
K276		65.0441554	31.7962767	qanat		361	
K277		65.0545080	31.7968079	qanat		377	
K278		65.0654729	31.7900415	qanat		1618	
K279		65.0730731	31.7901817	qanat		1230	

K280		65.2786769	31.7916280	qanat		1486	
K281		65.3579478	31.7923954	qanat		446	
K282		65.9783971	31.7893627	qanat		1121	
K283		65.6147238	31.7940409	qanat		658	
K284		66.0523330	31.7985420	qanat		573	
K285		64.8867797	31.7860791	qanat		757	
K286		64.8902330	31.7800063	qanat		1342	
K287		64.9286250	31.7800432	qanat		1680	
K288		65.0102202	31.7798366	qanat		1052	
K289		65.0416614	31.7855700	qanat		611	
K290		65.0486565	31.7868982	qanat		358	
K291		65.0517188	31.7848837	circular structure	0.52	79	79
K292		65.0603963	31.7798366	qanat		939	
K293		65.0633330	31.7778148	circular structure	0.27	61	57
K294		65.0664617	31.7881895	mound			
K295		65.1392710	31.7851215	qanat		239	
K296		65.2335589	31.7845756	structure	0.24	53	49
K297		65.3155002	31.7872500	qanat		1652	
K298		65.7648508	31.7831775	mound	1.79	164	113
K299		66.0383375	31.7800640	qanat		1640	
K300		66.0505920	31.7868073	qanat		1106	
K301		65.0369413	31.7788873	circular structure	0.79	93	93
K302		65.0573777	31.7698864	circular structure	0.38	69	69
K303		65.0657588	31.7718120	structure			
K304		65.5750355	31.7787877	qanat		667	
K305		65.5951178	31.7700819	qanat		908	
K306		65.8133376	31.7729150	qanat		579	
K307		65.8957952	31.7749918	qanat		1253	
K308		65.9242661	31.7721219	qanat		2445	
K309		65.9605426	31.7722325	qanat		2478	
K310		64.8899871	31.7671308	qanat		865	
K311		64.9198892	31.7627705	fortified town	80	1135	1048
K312		64.9465525	31.7619885	mound	0.09	37	37
K313		64.9482125	31.7600555	mound	0.11	35	33
K314		64.9556714	31.7639067	qanat		395	
K315		64.9702868	31.7680604	qanat		905	
K316		64.9981526	31.7688720	qanat		1167	
K317		65.1472353	31.7662381	qanat		1094	
K318		65.2951449	31.7623353	qanat		442	
K319		65.5424110	31.7655151	structure	0.07	29	29
K320		65.5750945	31.7662897	qanat		788	
K321		65.6078813	31.7628148	qanat		215	
K322		65.8172293	31.7617192	qanat		253	
K323		65.9462518	31.7614462	qanat		1102	
K324		65.9571857	31.7642719	qanat		1835	
K325		66.0071037	31.7609077	qanat		925	
K326		66.0081071	31.7605609	qanat		3110	
K327		66.0308306	31.7642572	qanat		1468	
K328		66.0325201	31.7655557	mound	0.09	31	31

K329		66.0331473	31.7659909	mound	0.06	30	30
K330		64.8932960	31.7564072	qanat		688	
K331		64.9027248	31.7559793	qanat		1243	
K332		64.9378430	31.7573221	qanat		1240	
K333		64.9364265	31.7526962	qanat		642	
K335		65.2991363	31.7591112	qanat		298	
K336		65.7219380	31.7569753	mound	0.57	101	69
K337		65.9195295	31.7501287	qanat		1118	
K338		65.9686803	31.7496123	qanat		3441	
K339		64.8955002	31.7445868	mound			
K340		64.9056228	31.7479266	qanat		1066	
K341		64.9173896	31.7440191	qanat		1891	
K342		65.1177084	31.7459102	qanat		1585	
K343		65.1189912	31.7440817	qanat		1648	
K344		65.5408325	31.7464631	qanat		596	
K345		65.9123971	31.7423152	qanat		1036	
K346		65.9559854	31.7475720	qanat		784	
K347		65.9657250	31.7399927	qanat		1466	
K349		66.1237400	31.7446155	qanat		386	
K350		65.5198957	31.7307619	qanat		319	
K351		65.5553002	31.7350750	qanat		517	
K352		66.0082332	31.7391485	qanat		1016	
K353		66.0373854	31.7299546	qanat		3733	
K354		66.0559576	31.7339212	qanat		2418	
K355		66.0650115	31.7329406	qanat		527	
K356		66.1196149	31.7333903	circular structure	0.67	92	87
K357		65.0854686	31.7201118	qanat		933	
K358		65.2519403	31.7240194	structure	0.21	41	40
K359		65.8768710	31.7240489	qanat		174	
K360		65.9552555	31.7258774	qanat		2323	
K361		65.9645084	31.7217559	structure			
K362		65.9619279	31.7247936	mound	0.35	60	59
K363		66.0175119	31.7247272	qanat		1779	
K364		66.1237842	31.7283252	qanat		334	
K365		65.2955511	31.7101339	qanat		1124	
K366		65.5309716	31.7137705	structure	0.1	33	33
K367		65.6017875	31.7099312	qanat		2503	
K368		65.9690059	31.7099239	qanat		985	
K369		65.9726535	31.7100123	qanat		939	
K370		66.1027823	31.7134315	qanat		1795	
K371		66.1186699	31.7180961	structure	0.41	68	63
K372		65.0687671	31.7033739	qanat		1851	
K373		65.0822056	31.7039633	qanat		991	
K374		65.4547340	31.6996974	qanat		647	
K375		65.6872079	31.7098905	mound	0.85	136	71
K376		65.9573855	31.7072234	qanat		1946	
K377		66.1202833	31.6999221	qanat		545	
K378		64.9261731	31.6941085	qanat		1133	
K379		65.0957170	31.6936518	mound	0.85	136	71

K380		64.9023356	31.6880533	qanat		774	
K381		64.9182690	31.6799209	qanat		1100	
K382		65.9649940	31.6972650	qanat		1590	
K383		65.9684783	31.6969703	qanat		1689	
K384		65.4475228	31.6868637	qanat		1375	
K385		65.7345967	31.6847274	qanat		481	
K386		65.7825075	31.6843444	mine			
K387		65.9485965	31.6835930	qanat		950	
K388		65.9568174	31.6821418	qanat		1331	
K389		66.0463774	31.6834899	qanat		807	
K390		64.8899360	31.6776913	qanat		678	
K391		64.9057949	31.6791752	qanat		754	
K392		64.9208179	31.6759865	qanat		541	
K393		64.9497077	31.6741455	qanat		1158	
K394		65.0741631	31.6750955	qanat		3238	
K395		65.4125711	31.6705002	structure	0.31	57	56
K396		64.9049185	31.6631949	qanat		1884	
K397		65.0334168	31.6600062	qanat		936	
K398		65.0436531	31.6636588	qanat		2340	
K399		65.0530425	31.6614495	qanat		2433	
K400		65.0586982	31.6605364	qanat		3442	
K402		65.0660698	31.6600209	qanat		3483	
K403		65.0769909	31.6650138	qanat		2164	
K404		65.0811002	31.6601166	qanat		2112	
K405		65.7536419	31.6658531	mine			
K406		65.8396663	31.6627976	qanat		1522	
K407		65.8552014	31.6693135	qanat		1299	
K408		65.8829068	31.6672888	qanat		494	
K409		65.8925002	31.6630995	qanat		3920	
K410		65.9349198	31.6617926	qanat		1571	
K411		66.0885033	31.6631105	qanat		297	
K412		64.8963979	31.6517280	qanat		763	
K413		64.9328574	31.6499904	qanat		925	
K414		64.9394837	31.6499389	qanat		1932	
K415		64.9836298	31.6578757	qanat		846	
K416		65.0826271	31.6499978	qanat		2335	
K417		65.8302827	31.6537011	qanat		1473	
K419		65.8781248	31.6516249	qanat		389	
K420		65.9292947	31.6539367	qanat		1133	
K421		65.9297660	31.6509623	qanat		868	
K422		65.9650695	31.6540030	qanat		2269	
K423		64.8817712	31.6458726	qanat		541	
K424		64.8871589	31.6400801	qanat		491	
K425		64.9171741	31.6393588	qanat		538	
K426		64.9867507	31.6482721	qanat		989	
K427		64.9965766	31.6490817	qanat		312	
K429		65.0408044	31.6416037	qanat		978	
K430		65.0855475	31.6446803	qanat		1909	
K431		65.7261078	31.6467816	mound	0.77	117	76

K432		65.8506858	31.6469067	qanat		1929		
K433		65.9091264	31.6399955	qanat		299		
K434		65.9472746	31.6401795	structure	0.32	71	55	
K435		65.9620761	31.6447428	qanat		1258		
K436		65.9793359	31.6475544	mound	0.22	60	47	
K437		64.8834125	31.6367956	mound	0.07	30	22	
K438		64.8904698	31.6341463	qanat		854		
K439		64.9236442	31.6345290	qanat		372		
K440		65.3499470	31.6332007	structure	0.13	39	39	
K441		64.8995053	31.6272991	qanat		477		
K442		64.9766279	31.6201254	qanat		339		
K443		64.9827715	31.6199489	qanat		1622		
K444		65.6615527	31.6288957	mound	0.48	89	60	
K445		65.6666809	31.6296683	mound	3.4	278	154	
K446		65.8460377	31.6352049	mound	0.35	66	55	
K447		65.8491279	31.6348333	qanat		506		
K448		65.9168842	31.6300729	qanat		892		
K449		65.9355505	31.6335016	qanat		1126		
K450		65.9755613	31.6307057	qanat		390		
K451		66.0166315	31.6308308	qanat		427		
K452		65.7261232	31.6229839	mound	2.1	190	138	
K453		65.8873176	31.6220973	qanat		1062		
K454		65.9051746	31.6210231	qanat		361		
K455		65.9124733	31.6199415	qanat		415		
K456		65.9687297	31.6233407	mound	0.34	63	61	
K457		65.9924066	31.6223033	qanat		412		
K458		66.0508997	31.6200519	qanat		2003		
K459		64.9755431	31.6151611	qanat		1381		
K460		64.9774411	31.6123804	qanat		1091		
K461		64.9838632	31.6098645	qanat		591		
K462		65.0149144	31.6100337	qanat		917		
K463		65.0166726	31.6100337	qanat		731		
K464		65.0534324	31.6163712	qanat		1381		
K465		65.0590747	31.6172761	qanat		1022		
K466		65.0614508	31.6153708	mound	0.06	26	26	
K467		65.0884046	31.6164742	qanat		863		
K468		65.8866775	31.6112071	qanat		1041		
K469		65.8942803	31.6176439	structure	0.06	26	26	
K470		65.9839176	31.6141055	qanat		440		
K471		66.0022203	31.6171510	qanat		743		
K472		66.0531264	31.6145910	qanat		570		
K473		66.0525379	31.6207704	mound	0.24	60	42	
K474		66.0582096	31.6176439	mound	0.66	153	77	
K475		66.0597618	31.6196669	mound	0.46	78	63	
K476		64.9783422	31.6097027	circular structure	0.33	48	48	
K477		64.9854632	31.6044576	qanat		238		
K478		64.9913262	31.6018461	qanat		178		
K479		64.9961447	31.6004410	qanat		327		
K480		65.5557732	31.6088052	mine				

K481		65.8456694	31.6067123	qanat		1126	
K482		65.8705118	31.6066608	qanat		390	
K483		65.9003897	31.6002792	circular structure	0.3	61	61
K484		66.0440302	31.6078195	qanat		645	
K485		65.0109310	31.5914810	qanat		232	
K486		65.0396061	31.5930699	qanat		494	
K487		65.0457340	31.5929670	qanat		695	
K488		65.0520825	31.5940778	qanat		1403	
K489		65.0629479	31.5900465	qanat		1024	
K491		65.6780694	31.5987753	mound	1.2	131	118
K492		65.7267966	31.5965654	mound	0.95	124	100
K493		65.7283667	31.5968816	mound	0.15	50	32
K494		65.8752063	31.5908330	qanat		1177	
K495		65.9353540	31.5955616	qanat		319	
K496		66.0046501	31.5946571	qanat		1317	
K497		64.9990565	31.5799382	qanat		656	
K498		64.9919760	31.5887099	circular structure	0.21	53	53
K499		65.0149529	31.5862872	qanat		887	
K500		65.0999271	31.5820374	structure	0.11	30	30
K501		65.5459072	31.5848388	qanat		1083	
K502		65.6722656	31.5888643	mound	0.39	80	65
K503		65.6839231	31.5888312	mound	0.27	55	50
K504		65.7951976	31.5858093	qanat		1144	
K505		65.8862450	31.5825962	qanat		1226	
K506		65.9824834	31.5855373	qanat		1457	
K507		64.8940706	31.5746276	qanat		383	
K508		64.8993049	31.5730691	qanat		2472	
K509		64.8995549	31.5697903	qanat		1891	
K510		64.9467518	31.5699079	qanat		1645	
K511		64.9494866	31.5699006	qanat		888	
K512		64.9815688	31.5733632	qanat		364	
K513		64.9961763	31.5700917	qanat		2453	
K514		65.0657073	31.5730029	qanat		214	
K515		65.6441865	31.5753848	qanat		291	
K516		65.7145666	31.5781049	mound	1.3	160	104
K517		65.7844799	31.5761126	qanat		603	
K519		66.0383070	31.5773403	mound	1.8	161	149
K520		64.9125304	31.5618837	qanat		1373	
K521		64.9169119	31.5616190	qanat		1754	
K522		64.9235577	31.5609721	qanat		2273	
K523		65.0051195	31.5686508	qanat		586	
K524		65.0069206	31.5631813	qanat		340	
K525		65.5410883	31.5628835	qanat		734	
K526		65.6714571	31.5662322	mound	0.37	75	48
K527		65.5860872	31.5648905	qanat		1270	
K528		65.6985255	31.5680847	circular structure	0.07	26	26
K529		65.7388120	31.5615713	circular structure	0.09	30	30
K530		65.7772754	31.5666438	mound	1.57	151	117
K531		64.9032784	31.5568810	qanat		562	

K532		64.9255463	31.5497794	qanat		855	
K533		64.9495050	31.5499338	qanat		1528	
K534		64.9548128	31.5500220	qanat		1450	
K535		64.9644434	31.5500661	qanat		1676	
K536		64.9928351	31.5490957	qanat		625	
K537		65.0102656	31.5504410	qanat		2440	
K538		65.0331216	31.5505954	qanat		1592	
K539		65.0560437	31.5551975	mound	0.71	93	88
K540		65.0585727	31.5572927	mound	0.09	36	36
K541		65.0885082	31.5565428	qanat		896	
K542		65.0952496	31.5554989	qanat		317	
K543		65.0982417	31.5547784	qanat		1041	
K544		65.1431302	31.5597481	structure	0.14	38	37
K545		65.1497613	31.5517275	qanat		786	
K546		65.1566939	31.5525068	qanat		1413	
K547		65.1688092	31.5501690	qanat		1851	
K548		65.1763446	31.5502646	qanat		1556	
K549		65.5960742	31.5596194	qanat		1951	
K550		65.6053740	31.5535985	qanat		1420	
K551		65.6174158	31.5511431	mound	0.66	95	73
K552		65.6599225	31.5546571	mound	0.72	104	72
K553		65.6826131	31.5529185	mound	1.14	135	105
K554		64.9763672	31.5450685	qanat		395	
K555		64.9867679	31.5420522	circular structure	0.49	75	75
K556		64.9880501	31.5416371	mound	0.05	31	23
K557		65.0028043	31.5400500	qanat		1148	
K558		65.0123050	31.5403218	qanat		603	
K559		65.0216954	31.5464939	qanat		927	
K560		65.0346127	31.5400279	qanat		1551	
K561		65.0387275	31.5403512	qanat		686	
K562		65.0426365	31.5400279	qanat		789	
K563		65.1892239	31.5414607	qanat		1630	
K564		65.1961969	31.5399471	qanat		888	
K565		65.2040663	31.5429009	qanat		1732	
K566		65.2253527	31.5420559	cluster of mounds			
K567		65.2367123	31.5399912	qanat		2159	
K568		65.5150815	31.5450979	qanat		492	
K569		65.8893534	31.5415122	qanat		1204	
K570		65.0950668	31.5370690	qanat		491	
K571		65.1104718	31.5365989	qanat		850	
K572		65.1250540	31.5374510	qanat		232	
K573		65.1484443	31.5391774	structure	0.2	50	50
K574		65.2064278	31.5338661	qanat		394	
K575		65.2472506	31.5335282	qanat		1509	
K576		65.2728227	31.5303546	qanat		1499	
K577		65.5357429	31.5325658	mound	0.32	66	66
K578		65.5610432	31.5323234	mound	1.05	116	105
K579		65.6034062	31.5304139	mound	1.03	129	95
K580		65.9576990	31.5372388	qanat		574	

K581		65.9603437	31.5318979	qanat		283	
K582		65.0790417	31.5252420	circular structure	0.19	36	36
K583		65.1306950	31.5278683	circular structure	0.51	74	74
K584		65.2146950	31.5259583	qanat		322	
K585		65.2173324	31.5256350	qanat		353	
K586		65.2231729	31.5237029	qanat		274	
K587		65.2266404	31.5236147	qanat		276	
K588		65.2484522	31.5211500	mound	2.78	267	111
K589		65.2469535	31.5243788	qanat		328	
K590		65.2513247	31.5228580	qanat		1303	
K591		65.2558796	31.5246138	qanat		243	
K592		65.2756417	31.5213740	mound	0.24	61	54
K593		65.2764939	31.5214328	mound	0.35	64	51
K594		65.2783489	31.5228286	qanat		1099	
K595		65.2834400	31.5201839	qanat		1726	
K596		65.2900371	31.5201398	qanat		1607	
K597		65.3785112	31.5258187	mound	0.46	75	75
K598		65.4024424	31.5245587	mound	0.4	70	70
K599		65.4944428	31.5205255	mound	0.65	109	92
K600		65.5181169	31.5231041	mound	0.88	96	90
K601		65.1899189	31.5148283	mound	0.2	41	41
K602		65.2203775	31.5195741	mound	0.77	121	83
K603		65.2901216	31.5163123	qanat		738	
K604		65.4334153	31.5193317	mound	0.14	38	38
K605		65.4559985	31.5137998	mound	0.2	45	45
K606		65.4648878	31.5111477	mound	0.25	50	50
K607		65.4641825	31.5169000	mound	0.27	50	50
K608		65.5384410	31.5130651	qanat		683	
K609		65.5394695	31.5126684	mound	0.41	56	51
K610		65.5691200	31.5146373	mound	0.41	73	60
K611		65.5704167	31.5145197	mound	0.42	79	58
K612		65.5888454	31.5103432	mound	1.09	112	111
K613		65.6273595	31.5169147	mound	0.32	55	46
K614		65.6272787	31.5158935	mound	0.23	50	44
K615		65.6327592	31.5167678	mound	1.69	140	138
K616		65.7959876	31.5176200	qanat		834	
K617		65.8270707	31.5164078	qanat		1430	
K618		65.8960544	31.5185162	qanat		1913	
K619		65.9349101	31.5172820	qanat		596	
K620		65.2331935	31.4999736	mound	0.16	47	47
K621		65.2922595	31.4997239	mound	0.7	87	71
K622		65.4750443	31.5031988	qanat		2465	
K623		65.5233550	31.5025155	mound	0.25	57	57
K624		65.5340405	31.5083523	mound	0.28	51	32
K625		65.6292255	31.5042273	mound	0.46	77	68
K626		65.6276534	31.5013181	mound	0.19	54	41
K627		65.6289757	31.5017001	mound	0.15	48	38
K628		65.2353070	31.4916616	mound	0.79	116	98
K629		65.5006149	31.4945215	qanat		3235	

K630		65.5185457	31.4930457	mound	0.21	61	38
K631		65.5365425	31.4982663	mound	0.09	52	39
K632		65.5364544	31.4961736	mound	0.19	60	41
K633		65.5565034	31.4949658	cluster of mounds			
K634		65.6122267	31.4971392	mound	0.65	119	66
K635		65.6954370	31.4905795	mound	0.38	63	61
K636		65.6971696	31.4926535	mound	0.67	92	82
K637		65.7775384	31.4920405	qanat		1476	
K638		65.7719588	31.4947862	qanat		211	
K639		65.8155167	31.4919230	qanat		1287	
K640		65.3816944	31.4815933	mound	0.84	107	97
K641		65.3906517	31.4866574	mound	0.36	70	62
K642		65.4504311	31.4821692	structure	0.27	53	51
K644		65.5268505	31.4861621	mound	0.37	67	67
K645		65.5797997	31.4814013	mound	1.34	140	128
K646		65.6185016	31.4838746	qanat		2438	
K647		65.7565579	31.4870052	qanat		1100	
K648		65.7813821	31.4806120	qanat		731	
K650		66.0560597	31.4807588	qanat		1386	
K651		65.3909071	31.4702110	mound	0.66	106	83
K652		65.4868495	31.4787476	mound	0.57	80	80
K653		65.4899250	31.4771034	mound	0.61	107	58
K654		65.4881414	31.4760317	mound	0.65	87	78
K655		65.4872826	31.4742628	mound	0.35	63	62
K656		65.4920316	31.4758189	mound	0.18	45	41
K657		65.5218930	31.4755528	mound	1.16	131	99
K658		65.5305763	31.4793403	mound	0.35	77	67
K659		65.5388486	31.4781916	cluster of mounds			
K660		65.7814720	31.4729691	qanat		1047	
K661		65.2351079	31.4683155	mound	0.26	71	41
K662		65.2370273	31.4658015	mound	0.7	150	74
K663		65.2523865	31.4679925	mound	0.85	114	92
K664		65.2643435	31.4608102	mound	0.46	67	65
K665		65.2637343	31.4684880	mound	1.57	135	121
K666		65.2968693	31.4668474	mound	0.62	92	77
K667		65.3224533	31.4674750	mound	0.78	97	86
K668		65.3477546	31.4650418	mound	0.31	57	53
K669		65.4043743	31.4600468	qanat		1050	
K670		65.5723892	31.4604065	qanat		3497	
K671		65.6290621	31.4668071	qanat		2733	
K672		65.6539029	31.4656657	mound	0.08	34	31
K673		65.6551287	31.4620397	mound	0.11	38	35
K674		65.7453835	31.4643275	qanat		1693	
K675		65.9103229	31.4625659	qanat		1476	
K676		65.9254362	31.4604666	qanat		677	
K677		65.9327469	31.4649881	qanat		2243	
K678		65.9549874	31.4592481	qanat		963	
K679		65.9606540	31.4573544	qanat		1972	
K680		65.9964076	31.4644082	qanat		1660	

K681		66.0411756	31.4637083	structure	0.11	33	33
K682		66.0653455	31.4670745	mine/querry			
K683		65.2978674	31.4543055	mound	1.64	171	130
K684		65.3026570	31.4529465	mound	0.29	61	59
K685		65.3038580	31.4531742	mound	0.37	72	72
K686		65.3174592	31.4572516	mound	3.43	197	179
K687		65.3337653	31.4585912	mound	0.47	73	64
K688		65.3345617	31.4570168	mound	0.39	70	64
K689		65.3484492	31.4589766	mound	0.21	57	47
K690		65.4174718	31.4540770	qanat		835	
K691		65.5172018	31.4589288	mound	0.44	77	75
K692		65.5360511	31.4575764	mound	0.1	36	36
K694		65.6091218	31.4521393	qanat		2163	
K695		65.6432974	31.4521833	qanat		3604	
K696		65.7399592	31.4553836	qanat		591	
K697		65.7503748	31.4542385	qanat		591	
K698		65.7763807	31.4529320	qanat		2476	
K699		65.3229588	31.4397428	mound	0.52	82	77
K700		65.3407984	31.4415620	mound	1.16	115	87
K701		65.3644696	31.4465757	mound	0.42	73	67
K702		65.5671551	31.4480520	mound	0.23	51	49
K703		65.5859612	31.4409055	qanat		1131	
K704		65.7451846	31.4481657	structure	0.67	87	75
K705		65.7880121	31.4470782	qanat		3460	
K706		65.9049159	31.4445108	qanat		364	
K707		65.4451836	31.4363072	mound	0.17	47	41
K708		65.4557569	31.4370332	mound	0.14	39	39
K709		65.5452074	31.4330126	mound	0.27	57	53
K710		65.5656169	31.4329062	mound	0.2	50	49
K711		65.5752680	31.4351668	qanat		1211	
K712		65.6278577	31.4308326	qanat		1660	
K713		65.6418888	31.4363805	fortress	2.32	163	150
K714		65.6583565	31.4373339	qanat		3109	
K715		65.6950762	31.4370992	qanat		650	
K716		65.7244255	31.4338064	qanat		1146	
K718		65.9079062	31.4329117	qanat		771	
K719		65.9258004	31.4331464	qanat		1502	
K720		65.9336034	31.4297582	qanat		847	
K721		65.4613250	31.4293714	mound	0.4	75	57
K722		65.5285251	31.4261959	mound	0.08	39	33
K723		65.5558687	31.4282512	qanat		818	
K724		65.5840886	31.4272538	qanat		1201	
K725		65.5963028	31.4249657	mound	0.14	49	35
K726		65.5981105	31.4252810	mound	0.07	33	23
K727		65.7261415	31.4283245	qanat		2043	
K728		65.9163911	31.4269761	qanat		764	
K729		65.9372288	31.4593380	mound	1.8	143	136
K730		65.9509005	31.4526852	mound	0.8	102	99
